# Physically aligned forest fire risk prediction: A deep learning framework coupling fuel and climate multivariate factors

**DOI:** 10.1371/journal.pone.0355829

**Published:** 2026-09-01

**Authors:** Bojie Chen, Anping Zeng, Yu Xie, Xinjie Wang, Lihong Zhu, Qina Xie, Guoqing Long

**Affiliations:** 1 School of Computer Science and Technology, Yibin University, Yibin, China; 2 School of Business English, Chengdu International Studies University (Yibin Campus), Yibin, China; Oregon State University, UNITED STATES OF AMERICA

## Abstract

Existing forest fire risk prediction methods often focus on single-element analysis, which makes it difficult to effectively capture the underlying mechanisms of the “fuel-climate” interaction. This paper proposes a physically aligned prediction framework named FWI-MSNet. Using 18 years of synchronized observation data from the Huitong Ecological Station in China, the framework constructs a multi-scale feature system, selecting 21 physically relevant key features, including core indicators of the Forest Fire Weather Index (FWI). A parallel multiscale 1-Dimensional Convolutional Neural Network (1D-CNN) extracts dynamic features across multiple temporal scales (from daily to seasonal). Subsequently, a synergistic mechanism integrating Gated Recurrent Units (GRU) and Transformer achieves deep integration of temporal evolution and global correlation. Experimental results demonstrate that compared to seven baseline models (including XGBoost, LSTM, CNN, and four ablation variants), the mean of RMSE, MAE, and MAPE decreases by 56.1%, and R^2^ improves from 0.223 (baseline average) to 0.9251. Furthermore, it accurately captures the trajectory of fire risk evolution in the 2020 catastrophic forest fires in Australia. This study provides a valuable reference for forest fire risk prediction.

## 1. Introduction

**Fig 1:**
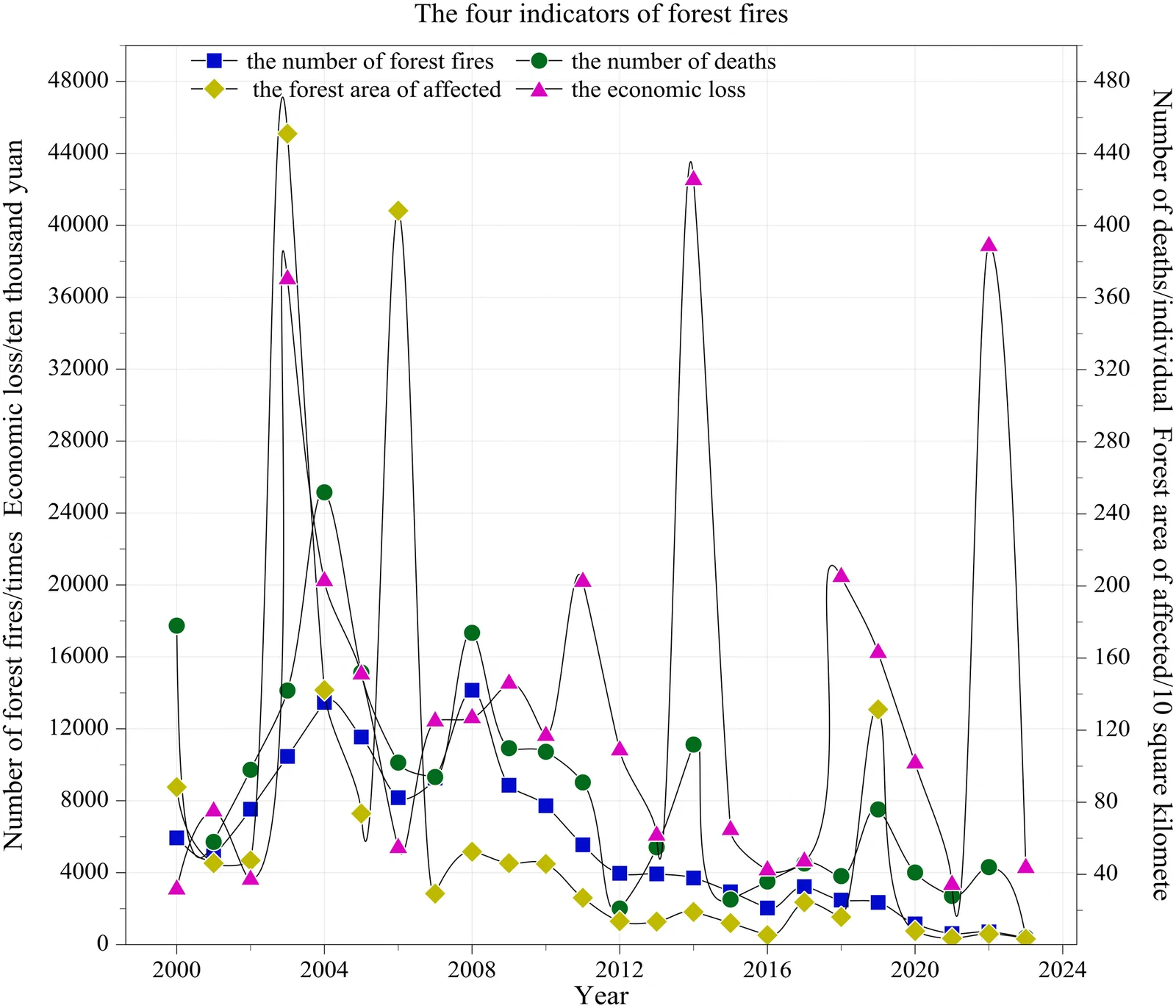
Key fire indicators. From 2000 to 2023, the frequency of forest fires and the direct economic losses they caused in China showed an upward trend, while the area of forest affected decreased significantly. This trend reflects that, although the burned area per single fire has been effectively controlled, the increasing frequency of fires and the associated economic losses have not diminished correspondingly, suggesting that fire risk may be more influenced by the interactive effects of fuel accumulation and climatic conditions.

Forest fires, driven by the complex interplay of meteorological conditions, fuel characteristics, and anthropogenic activities [1], have become a major global concern. As climate change intensifies, the frequency, intensity, and duration of the wildfire season have risen significantly, posing severe threats to ecological balance, human safety, and socioeconomic development [2–4]. Data from the China Statistical Yearbook (2000–2023) corroborate this trend, revealing notable fluctuations and periodic peaks in key fire indicators (Fig 1).

Traditional early warning systems primarily rely on physical sensor technologies within Wireless Sensor Networks (WSNs), utilizing temperature, humidity, and smoke detectors for real-time monitoring [5–7]. However, the large-scale deployment of WSNs presents persistent challenges related to energy consumption, network lifespan, and real-time responsiveness, which limit their predictive capability for large-area risk assessment [8].

Consequently, researchers have increasingly turned to data-driven approaches. Among these, machine learning and multi-source data fusion have driven recent progress in forest fire risk assessment. Abbas Khurram [9] et al. used random forest and gradient boosting machines to confirm temperature and relative humidity as critical factors. Jiajun Chen [10] et al. built a risk model using exponentially weighted decay and support vector machine regression, achieving high test accuracy. Diana Škurić Kuraži [11] et al. proposed a new soil-based fire risk index that outperforms traditional weather indices. Although these methods demonstrate utility, their predictive accuracy is often constrained by a limited capacity to model complex nonlinear spatiotemporal dependencies.

In contrast, deep learning approaches have been increasingly adopted for forest fire risk prediction due to their ability to model complex spatiotemporal patterns. Recent comparative studies have also evaluated the performance of CNN, LSTM, GRU, and Transformer architectures for time series forecasting in various domains [12]. Mohammad Marjani [13] et al. developed a CNN-BiLSTM hybrid model for near-real-time wildfire spread prediction, achieving an Intersection over Union (IoU) of 0.58 and an F1 score of 0.73, demonstrating the effectiveness of combining convolutional feature extraction with sequential modeling. Xufeng Lin [14] et al. proposed a forest fire prediction model based on LSTNet, which captures both short-term and long-term temporal dependencies; the model achieved a high accuracy of 0.941, highlighting the importance of utilizing spatial background information and the periodic nature of fire-related factors. More recently, Xinyu Miao [15] et al. introduced a window-enhanced Transformer model for time series prediction, achieving strong performance (ACC = 91.56%, RMSE = 0.37, MAE = 0.05) by effectively leveraging spatial context and periodic patterns in forest fire drivers. Despite these advances, most deep learning models treat fire prediction as a purely data-driven task, with limited integration of the physical principles governing fire behavior—such as the multi-scale temporal dynamics embedded in the Fire Weather Index (FWI) system.

Furthermore, researchers have attempted to enhance physical interpretability by improving traditional indices. Santos L C [16] et al. developed an enhanced FWI (FWIe), which correlates indices with satellite-detected fires via fire radiative power, thereby improving risk assessment. Binita Kumari [17] et al. evaluated risk using various triggering factors, finding that GIS-based risk areas closely match historical fire occurrences. These efforts highlight the growing recognition of integrating physical knowledge with data-driven methods. However, most existing approaches either treat physical indices as mere input variables without exploiting their inherent multi-scale temporal structure, or rely on static spatial risk mapping that fails to capture the dynamic coupling between fuel and climate factors across different time scales.

Despite these significant efforts, several critical research gaps remain. First, most existing deep learning models [18] treat fire prediction as a purely data-driven task, often overlooking the inherent multi-scale temporal structure of the physical processes governing fire behavior [19] (e.g., daily weather-driven responses vs. seasonal soil moisture accumulation). Second, although the Fire Weather Index (FWI) system [20] provides a well-established physical framework for characterizing fire danger, its potential to serve as a physically aligned organizational principle for structuring multi-source features—rather than merely an additional input variable—remains largely untapped [21]. Third, existing architectures struggle to simultaneously capture the multi-scale temporal patterns [22] (from daily to seasonal variations) and long-term dependencies inherent in the coupled fuel-climate system [23], limiting their ability to align these interacting physical processes.

To bridge these gaps, this paper proposes FWI-MSNet, a novel physically aligned deep learning framework for the synchronous analysis of fuel-climate coupling. This study uses empirical data from the China Ecological Science Data Center (https://www.nesdc.org.cn/). The novelty of this framework lies in three aspects: physically aligned feature organization based on the FWI system‘s multi-scale temporal structure; explicit physical mapping between CNN kernel sizes and FWI component time scales; and a GRU-Transformer collaborative mechanism for temporal-global fusion. These design choices translate into three main contributions:

1. Physically Aligned Feature Organization: Unlike conventional black-box models that treat all inputs uniformly, our framework uses the Fire Weather Index (FWI) system as a physically meaningful basis for feature engineering. By organizing input features according to the FWI’s inherent multi-scale structure (short-term, medium-term, long-term), the learning process aligns with the physical principles of fire dynamics, leading to more consistent and interpretable predictions.
2. Multi-Scale Feature Extraction: We design parallel multi-scale 1D-CNN branches with kernel sizes aligned to the physical timescales of fire risk formation (daily, monthly, and quarterly). This enables the model to capture the diverse temporal patterns inherent in meteorological and fuel data—from short-term weather-driven responses to long-term drought accumulation—ensuring that features across all relevant physical scales are represented for subsequent fusion.
3. Sequential Modeling with Temporal-Global Fusion: We integrate a GRU-Transformer hybrid mechanism to model both the temporal evolution and global contextual dependencies within the multi-scale features. The GRU layer captures mid-term sequential patterns, while the Transformer encoder extracts cross-period correlations, enabling a comprehensive representation of the coupled fuel-climate dynamics for FWI prediction.

Experimental results on real-world datasets demonstrate that FWI-MSNet achieves superior performance compared to existing methods, thereby providing a more reliable scientific foundation for forest fire prevention and management.

The terminology and abbreviations used in this paper are listed (Table 1).

**Table 1:** The terminology and abbreviations.

Terminology	abbreviation
Fire Weather Index	FWI
1-Dimensional Convolutional Neural Network	1D-CNN
Gated Recurrent Unit	GRU
Fine Fuel Moisture Code	FFMC
Duff Moisture Code	DMC
Drought Code	DC
Buildup Index	BUI
Initial Spread Index	ISI

FWI and its sub-indices FFMC, DMC, DC, ISI, and BUI are used to characterize fuel moisture and fire behavior potential, serving as core physical quantities for fuel‑climate coupling. 1D‑CNN and GRU are employed for multi‑scale feature extraction and temporal dependency modeling, respectively. These abbreviations are used throughout the model construction and feature selection process, providing a clear variable basis for physically aligned deep learning prediction.

## 2. Methodology

**Fig 2:**
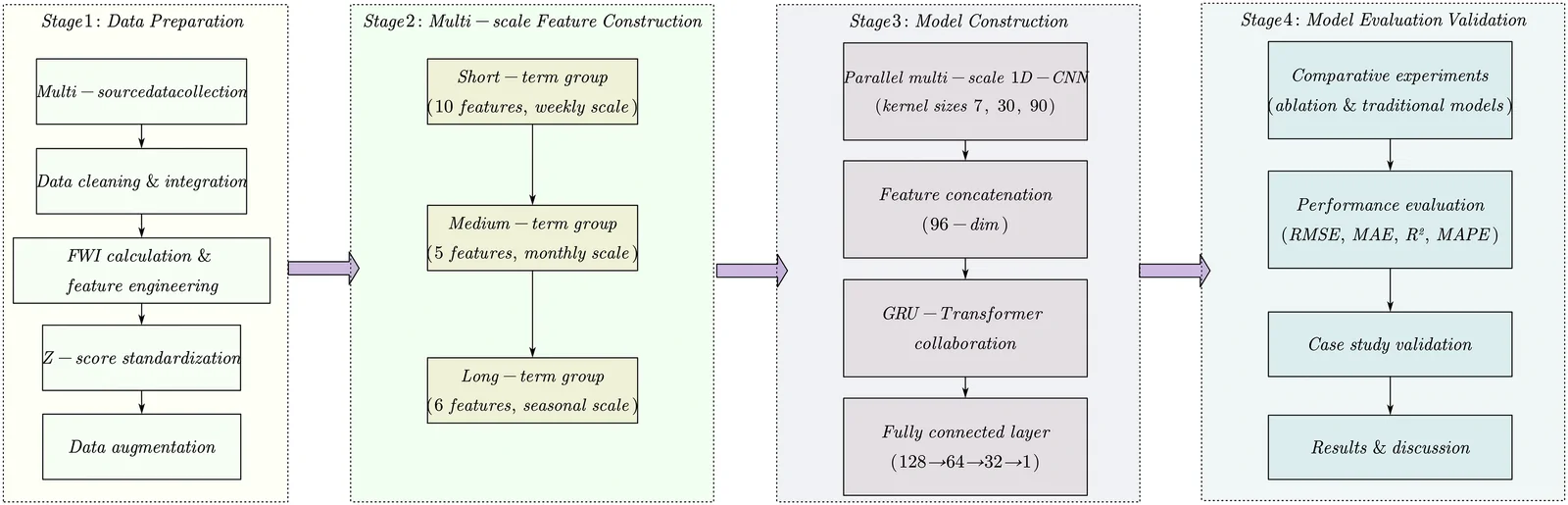
Overall workflow of this study. The workflow consists of four main stages: (1) Data Preparation, including data cleaning, FWI calculation, standardization, and augmentation; (2) Multi-scale Feature Construction, where 21 features are organized into three groups (short-term, medium-term, long-term) based on the FWI system’s temporal structure; (3) Model Construction, consisting of parallel multi-scale 1D-CNN, feature concatenation, GRU-Transformer collaboration, and fully connected output layer; and (4) Model Evaluation & Validation, including ablation experiments, comparisons with traditional models, and case study validation. Detailed parameter settings are provided in Tables 2–5 and Section 4.1.

The FWI-MSNet framework consists of four main stages(Fig 2): data preparation, multi-scale feature construction, model construction, and model evaluation. First, multi-source datasets are cleaned, integrated, and normalized, followed by data augmentation. Second, 21 key features are organized into three groups according to the FWI system’s multi-scale temporal structure. Third, a parallel multi-scale 1D-CNN with kernel sizes of 7, 30, and 90 days extracts features at daily, monthly, and seasonal scales, which are then fused and processed by a GRU-Transformer collaborative mechanism. Fourth, the model is trained and evaluated through ablation experiments, comparisons with traditional models, and a case study on the 2020 Australian wildfires. The detailed methodology is elaborated in the following sections.

**Table 2:** The specific parameters of data preprocessing.

Parameter Category	Parameter Name	Parameter Value	Description
Data Augmentation	base_seq_length	6	Base sequence length (6 months)
	seq_lengths	[3, 4, 5, 7, 8]	Multi-scale sampling sequence lengths
	noise_levels	[0.01, 0.02]	Standard deviation for noise augmentation
	stride	1	Sliding window stride
Data Split	train_ratio	0.8	Proportion of training set
	test_ratio	0.2	Proportion of test set
Normalization	scaler	StandardScaler	Normalization method

**Table 3:** Multi-scale feature grouping.

Characteristic group	Time scale	Representative input characteristics
Short-term-Climate Driving Group	Week	FFMC
Medium-term-Ecological Hydrological Driving Group	Month	DMC
Long-term – Soil Driving Group	Quarter	DC

**Table 4:** Parameter configuration of multi-scale one-dimensional convolutional layer.

Path name	The size of the convolutional nucleus	Feel the wild	Corresponding to physical processes	Main input feature source	Output the number of feature diagrams
Short-term path	7	7 days	Weather-driven quick response	Climate driven group	32
Medium-term path	30	A month	The process evolution of ecological hydrology	Hydrological driven Group	32
Long-term Path	90	A quarter	Climate-scale Drought Accumulation	Soil driven Group	32

**Table 5:** The parameter configuration of GRU-Transformer.

Parameter Category	Parameter Name	Parameter Value	Description
Input Parameters	input_dim	96	Input feature dimension (Multi-scale CNN output: 32 * 3 = 96)
	seq_length	6	Sequence length (monthly data, 6 months)
	batch_size	32	Batch size
Multi-scale CNN Parameters	kernel_sizes	[7, 30, 90]	Convolution kernel sizes (short-term/ medium-term/ long-term)
	out_channels	32	Output channels per path
	padding	‘same’	Preserve sequence length
	activation	ReLU	Activation function
GRU Layer Parameters	hidden_dim	128	GRU hidden layer dimension
	num_layers	1	Number of GRU layers (original paper value)
	dropout	0.1	Dropout rate between GRU layers
	bidirectional	FALSE	Unidirectional GRU
	batch_first	TRUE	Input tensor shape as (batch, seq, features)
Transformer Parameters	nhead	8	Number of multi-head attention heads
	num_layers	2	Number of Transformer encoder layers
	d_model	128	Model dimension (consistent with GRU hidden dimension)
	dim_feedforward	512	Feedforward network dimension (d_model * 4)
	dropout	0.1	Dropout rate for Transformer sublayers
	activation	ReLU	Activation function (original paper value)
	batch_first	TRUE	Input tensor shape as (batch, seq, features)
Output Layer Parameters	fc_layers	[128, 64, 32, 1]	Fully connected layer structure
	dropout	0.1	Dropout rate in output layer
	activation	ReLU	Activation function

### 2.1. Forest Fire Risk Weather Index

The FWI system is the physical core of this model, converting meteorological data into indices that reflect fuel moisture [24] and fire behavior [25]. Its chain includes three moisture and two behavior indices [26,27], leading to the final FWI [28]. These indices characterize drying conditions across time scales [29], providing a physical basis for multi-scale feature grouping. The mathematical expressions [30–32] are as follows:


$$\begin{array}{c} FFMC=\frac{59.5\times (250-M)}{147.2+M} \end{array}$$ (1)

$$\begin{array}{c} DMC=P_{e}+100\times [1.894(T+1.1)(100-H)L\times 10^{-6}] \end{array}$$ (2)

$$\begin{array}{c} DC=V+0.5\times [0.36(T+2.8)(100-H)+L_{f}] \end{array}$$ (3)

$$\begin{array}{c} BUI=\left\{ \begin{array}{c} @l@\text{\textbackslash{}hspace\{1em}\;\;\}\frac{0.8\times DMC\times DC}{DMC+0.4\times DC}\text{\textbackslash{}hspace\{1em}\;\;\}\;when\text{\textbackslash{}hspace\{0.5em}\;\}DMC\leq 0.4\times DC\\ DMC-(1-\frac{DMC}{DMC+0.4\times DC})\times [0.92+(0.0114\times DMC{)}^{1.7}]\text{\textbackslash{}hspace\{1em}\}otherwise \end{array}\right.\text{\textbackslash{}hspace\{1em}\} \end{array}$$ (4)

$$\begin{array}{c} f\left(D\right)=\left\{ \begin{array}{c} @l@0.626\times BUI^{0.809}+2\text{\textbackslash{}hspace\{1em}\}when\text{\textbackslash{}hspace\{0.33em}\}BUI\leq 80\\ \frac{1000}{25+108.64\times e^{-0.023\times BUI}}\text{\textbackslash{}hspace\{1em}\;\}\;otherwise \end{array}\right. \end{array}$$ (5)

$$\begin{array}{c} ISI=0.208\times e^{0.05039U}\times 91.9\times e^{-0.1386m}\times (1+\frac{m^{5.31}}{4.93\times 10^{\text{7}}}) \end{array}$$ (6)

$$\begin{array}{c} FWI=\left\{ \begin{array}{c} @l@\sqrt{ISI\times f\left(D\right)}\text{\textbackslash{}hspace\{1em}\;\;\}\;when\text{\textbackslash{}hspace\{0.33em}\}\sqrt{ISI\times f\left(D\right)}\leq 1\\ e^{\left[2.72\times (0.434\times ln\left(\sqrt{ISI\times f\left(D\right)}\right){)}^{0.647}\right]}\text{\textbackslash{}hspace\{1em}\}otherwise \end{array}\right. \end{array}$$ (7)

Among them, M is the humidity percentage of fine fuels; $P_{e}$ is the rainfall correction term of DMC; V is the rainfall correction item of DC; L is the daily length coefficient; $L_{f}$ is the daily length correction factor; m is the converted humidity index; f(D) is the availability function of combustible materials; T is the temperature (°C); U is the relative humidity (%); U is the wind speed (km/h).

### 2.2. One-dimensional convolutional neural network

1D-CNN, a one-dimensional convolutional neural network [33], processes sequential data [34]. Its parallel configuration extracts multi-scale temporal features [35]. With different-sized convolution kernels [36], it captures local patterns from weather-driven responses to soil drought conditions [37], enabling hierarchical representation of physical grouping features. The mathematical expressions [38] are as follows:


$$\begin{array}{c} y_{j}=\sum_{k=0}^{K-1}x_{j+k}•w_{k}+b \end{array}$$ (8)

Among them, $y_{j}$ represents the value of the output feature graph at position j; x is the input timing sequence; j is the weight of the convolutional kernel; K is the size of the convolutional kernel; b is the bias value; k is the index inside the convolutional kernel, from 0 to k − 1; j is the index of the output sequence.

### 2.3. Gated Recurrent Unit

GRU, a gated recurrent unit structure [39], is an efficient recurrent neural network variant [40]. Its update and reset gates alleviate gradient issues in long sequences [41] and model temporal dependencies [42]. In this model, the GRU layer models the evolution of multi-scale features, capturing mid-term memory and continuous patterns in environmental dynamics [43]. The mathematical expressions [44] are as follows:


$$\begin{array}{c} Z_{t}=\sigma \left(w_{z}\left[h_{t-1},x_{t}\right]+b_{z}\right) \end{array}$$ (9)

$$\begin{array}{c} R_{t}=\sigma \left(w_{r}\left[h_{t-1},x_{t}\right]+b_{r}\right) \end{array}$$ (10)

$$\begin{array}{c} H_{t}=T\left(w_{h}\left[r_{t}h_{t-1},x_{t}\right]+b_{h}\right) \end{array}$$ (11)

$$\begin{array}{c} h_{t}=\left(1-z_{t}\right)h_{t-1}+Z_{t}H_{t} \end{array}$$ (12)

In the formula: σ for the Sigmoid activation function, the output value is between [0, 1]; T for the hyperbolic tangent activation function, the output value is between [−1, 1]; $x_{t}$ for the input vector of the current moment; $h_{t-1}$ for the hidden state of the current moment; $z_{t}$ for the output of the update gate; $R_{t}$ for the loss of the reset gate Out; $w_{z}$ 、 $w_{r}$ and $w_{h}$ are the weighted matrix; $b_{z}$ 、 $b_{r}$ and $b_{h}$ are the bias term; $H_{t}$ is the candidate hidden state.

### 2.4. Transformer

The Transformer’s self‑attention mechanism computes correlations between all sequence elements [45]. Placed after GRU, it extracts global contextual dependencies, enabling the model to capture both local temporal evolution and cross‑period fire‑risk connections for a comprehensive view of global risk patterns.

The core computational mechanisms [46] of the Transformer encoder include scaled dot-product attention, multi-head attention, and feed-forward networks [47], whose mathematical formulations and functions are described below. In addition, to compensate for the Transformer’s inherent inability to perceive sequence order, sinusoidal position encoding is added to the input embeddings.

Scaled dot-product attention is used to compute the correlation weights between queries and keys, and then perform a weighted sum of the values. The mathematical expressions [48] are as follows:


$$\begin{array}{c} Attention\text{(}Q,K,V)=softma\text{x(}\frac{QK^{T}}{\sqrt{d_{k}}})V \end{array}$$ (13)

Multi-head attention projects the input into multiple subspaces and performs attention in parallel, enabling the model to jointly capture information from different representational subspaces. The mathematical expressions [48] are as follows:


$$\begin{array}{c} MultiHead\left(Q,K,V\right)=Concat(head_{\text{1}},...,head_{h})W^{O} \end{array}$$ (14)

$$\begin{array}{c} head_{i}=Attention\left(QW_{i}^{Q},KW_{i}^{K},VW_{i}^{V}\right) \end{array}$$ (15)

where $Q,K,V\in R^{n*d_{k}}$ denote the query, key, and value matrices, n is the sequence length, and $d_{k}$ is the dimension of each attention head.

Positional encoding is used to inject position information into the sequence to compensate for the Transformer’s inherent inability to perceive order. In this paper, sinusoidal functions are adopted to generate absolute positional encodings. The mathematical expressions [48] are as follows:


$$\begin{array}{c} PE_{\left(pos,2i\right)}=sin\left(\frac{pos}{10000^{2i/d}model}\right) \end{array}$$ (16)

$$\begin{array}{c} PE_{\left(pos,2i+1\right)}=cos\left(\frac{pos}{10000^{2i/d}model}\right) \end{array}$$ (17)

The feed-forward network applies a nonlinear transformation to the representation at each position, enhancing the model’s expressiveness. The mathematical expressions [48] are as follows:


$$\begin{array}{c} FFN\left(x\right)=ReLU(xW_{\text{1}}+b_{\text{1}})W_{\text{2}}+b_{\text{2}} \end{array}$$ (18)

### 2.5. Improved model

**Fig 3:**
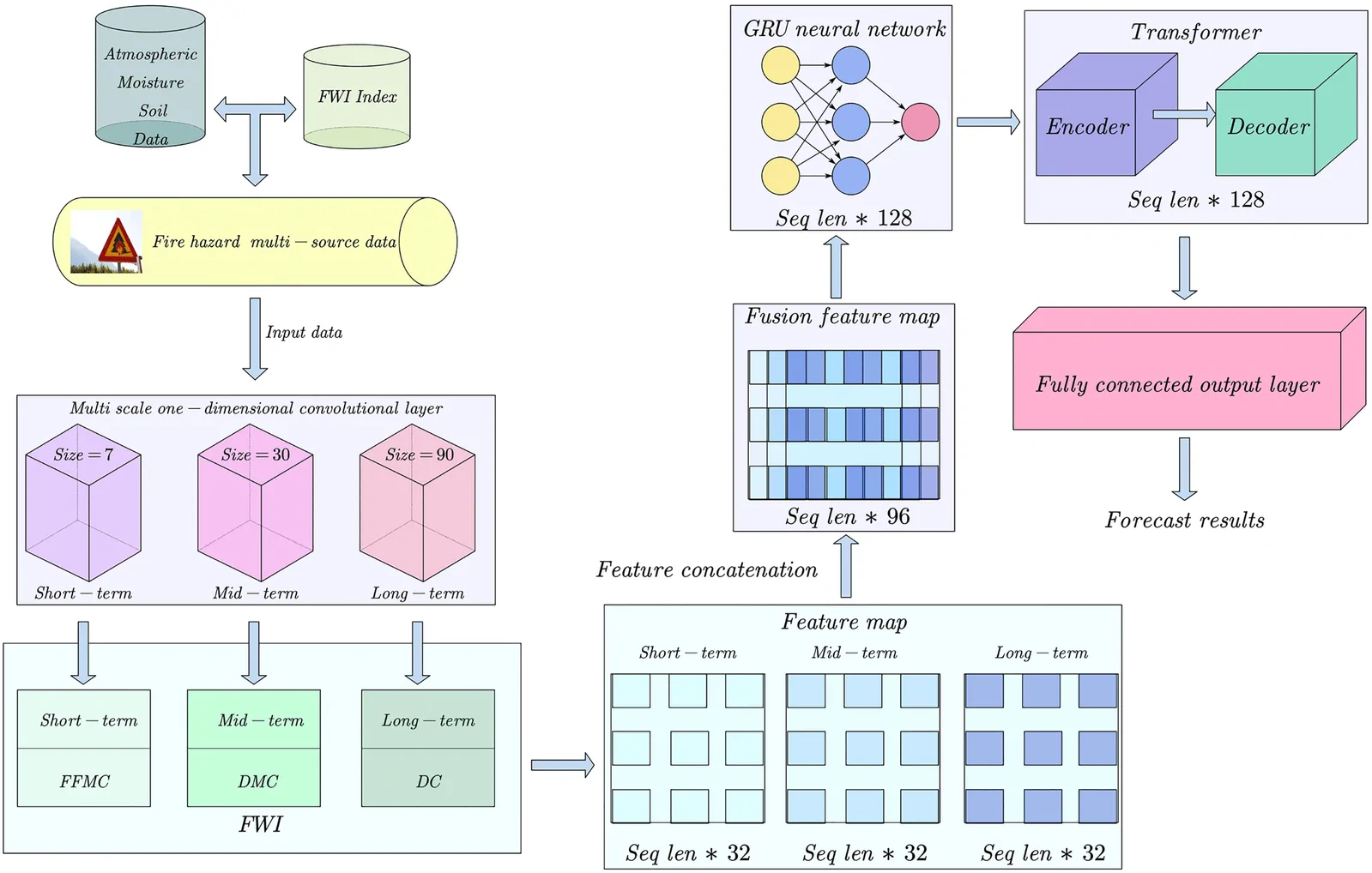
FWI‑MSNet model. The input layer integrates multi-source fire risk data, including atmospheric humidity, soil data, and the FWI. Parallel multi-scale 1D-CNN channels with kernel sizes of 7, 30, and 90 extract short-term, medium-term, and long-term dynamic features at daily, monthly, and seasonal scales, respectively, to capture the multi-temporal-scale response of fuel–climate coupling. Subsequently, a GRU and a Transformer collaboratively fuse temporal evolution features with global contextual features to generate a fused feature map, and the prediction result is output through a fully connected layer. This framework provides a clear computational path for interpreting the multi-scale driving mechanisms of fire risk.

FWI‑MSNet (Fig 3) addresses multi‑scale fire‑risk capture limitations with a physically aligned framework that uses parallel 1D‑CNN channels for daily‑to‑quarterly feature extraction. GRU and Transformer [49] fuse features temporally and globally.

The proposed FWI-MSNet framework is implemented using Python 3.9 with PyTorch 1.10 as the deep learning backend. The implementation leverages the following libraries: NumPy for numerical computations, Pandas for data manipulation, scikit-learn for data preprocessing and evaluation metrics. The complete source code is available at the accompanying GitHub repository (https://github.com/CBJYB/FWIMSNet-Forest-Fire-Risk-Prediction-Framework). The overall execution flow of the model mainly includes the following steps.

1. Data Preprocessing: standardization and data augmentation
2. Construction of multi-scale input features
3. Model Construction:
  Multi-scale Feature Extraction Module
  GRU-Transformer Collaborative Working Mechanism
  Output Layer Construction
4. Model Training and Evaluation
5. Model Prediction and Evaluation

#### 2.5.1. Data preprocessing.

Normalization (Table 2) is performed using `sklearn.preprocessing.StandardScaler`, which ensures that each feature has zero mean and unit variance, thereby eliminating the influence of different scales. Data augmentation is implemented through sliding window sampling combined with noise injection. The base sequence length is set to 6 months with a sliding stride of 1, and multi-scale sequences of varying lengths are introduced to enrich sample diversity. Gaussian noise with standard deviations of 0.01 and 0.02 is applied to enhance the data, enabling better fitting of random fluctuations in the observed data.

The base sequence length is set to 6 months to cover a complete seasonal cycle of fuel moisture variation, consistent with the multi-scale temporal structure of the FWI system (Section 2.1). Multi-scale sequence lengths of 3, 4, 5, 7, and 8 months are introduced to enrich sample diversity by capturing both shorter and longer seasonal patterns. The stride is set to 1 to maximize sample diversity from the limited time series. Gaussian noise with standard deviations of 0.01 and 0.02 is added to improve model robustness against observation errors. The 80/20 chronological split ensures that training and test sets are strictly separated in time, preventing any future information from leaking into the training process.

#### 2.5.2. Construction Of multi-scale input features.

This model groups features into three clear sets based on their physical processes and time scales [50]. It merges raw observations with calculated FWI indices [51] (Table 3) into a comprehensive feature pool that includes direct measurements like temperature and solar radiation, as well as physical simulation indices such as FFMC and DMC, using Pandas for data loading and scikit-learn’s StandardScaler for normalization.

The short-term (weekly) climate-driven group, represented by FFMC, captures the rapid response of fuel moisture to meteorological factors; the medium-term (monthly) eco-hydrological group, centered on DMC, reflects the seasonal moisture balance of the duff layer; the long-term (seasonal) soil-driven group, indicated by DC, characterizes the cumulative effect of deep-layer soil drought. This explicit grouping enables the model to extract features at time scales that match the fuel-climate coupling mechanism, providing a physical basis for the subsequent design of the multi-scale 1D-CNN kernel sizes (7, 30, and 90 days) and enhancing the model’s interpretability and generalization ability.

#### 2.5.3. Multi-scale feature extraction module.

This module consists of three parallel one-dimensional convolutional layers (Table 4), each focusing on a specific time scale. One-dimensional convolution can effectively capture local dependencies by sliding along the temporal dimension, and its receptive field size is directly determined by the size of the convolution kernel. This module uses torch.nn to implement three parallel 1D convolutional layers, with Conv1d padding to maintain sequence length. Each convolution is followed by batch normalization (nn.BatchNorm1d) and ReLU activation (nn.ReLU), with dropout (nn.Dropout) for regularization.

The kernel sizes (7, 30, and 90 days) of the three parallel 1D‑CNN channels are explicitly mapped to three physical processes: weather-driven dynamics, eco-hydrological succession, and climate-scale drought accumulation, respectively. This design enables the model to extract features with receptive fields that match the fuel‑climate coupling mechanism: the short-term path captures the rapid response of fuel moisture to meteorological disturbances at the weekly scale, the medium-term path reflects the moisture balance of the duff layer at the monthly scale, and the long-term path characterizes the drought memory of deep-layer soil at the seasonal scale. All output feature maps have 32 dimensions, ensuring balanced weighting of features from different scales during subsequent fusion. This parameter configuration embeds domain priors into the network structure, reducing reliance on black-box learning.

The mathematical expressions [52] for the one-dimensional convolution operation on each path are as follows:


$$\begin{array}{c} Z_{\text{i}}=RELU\text{(}Conv1D\left(X_{i},W_{i}\right)+b_{i}) \end{array}$$ (19)

Among them, $X_{i}$ is the input feature sequence of the i-path; $W_{i}$ and $b_{i}$ is the trainable weight and bias of the path convolution kernel; Conv1D represents a one-dimensional convolution operation; RELU for the activation function, a nonlinear transformation is introduced; $Z_{\text{i}}$ ∈R^T*32^ is the feature diagram of the output of the path.

The three parallel paths output feature maps, each with a dimension of 32. In order to integrate information from all scales, it is necessary to concatenate them on the feature dimension (the second dimension). The mathematical expression [53] is shown below.


$$\begin{array}{c} Z_{fused}=[Z_{short};Z_{mid};Z_{long}] \end{array}$$ (20)

[;] represents the splicing operation. The fusion feature tensor $Z_{fused}$ ∈R^T*96^ also contains multi-scale information from weather transients, hydrological trends and climate background, providing a rich feature basis for subsequent time series modeling.

**Fig 4:**
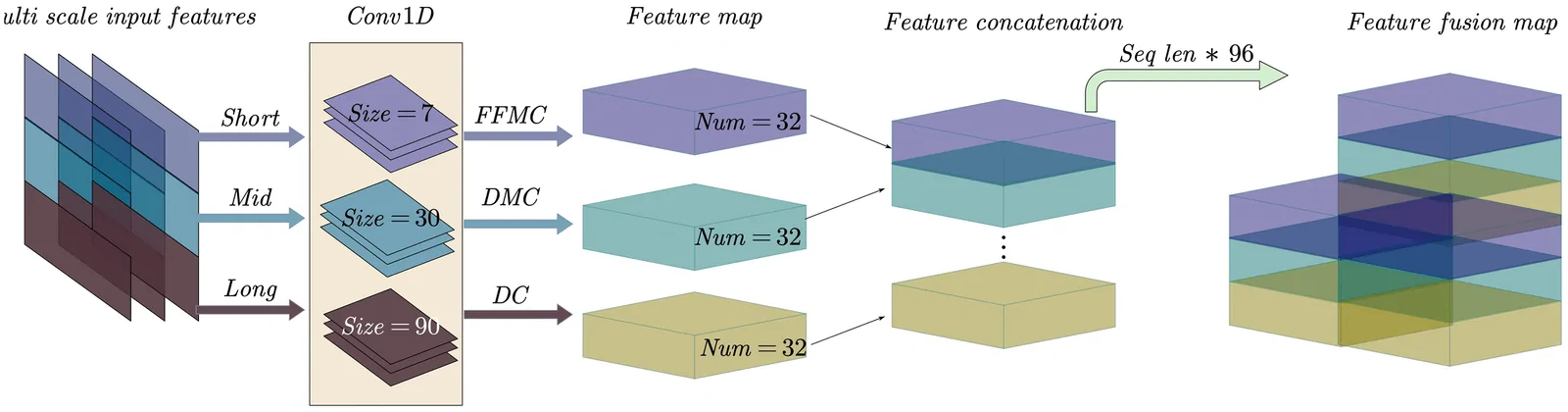
The feature extraction module. The kernel sizes 7, 30, and 90 correspond to the inherent variation periods (daily, monthly, and seasonal) of FFMC, DMC, and DC, respectively. This explicit mapping enables the network to extract features at time scales that match the physical mechanisms of fuel moisture, rather than blindly learning arbitrary temporal patterns. The feature maps output by the three parallel channels are concatenated to form a multi-scale representation, preserving cross-scale correlation information for the subsequent GRU-Transformer fusion.

The feature extraction module (Fig 4) processes short‑, medium‑, and long‑term features through three parallel 1D‑CNN channels using kernel sizes 7, 30, and 90 to extract patterns corresponding to FFMC, DMC, and DC dynamics, respectively. The resulting feature maps are concatenated along the feature dimension to form a unified multi‑scale representation.

#### 2.5.4. GRU transformer collaborative working mechanism.

**Fig 5:**
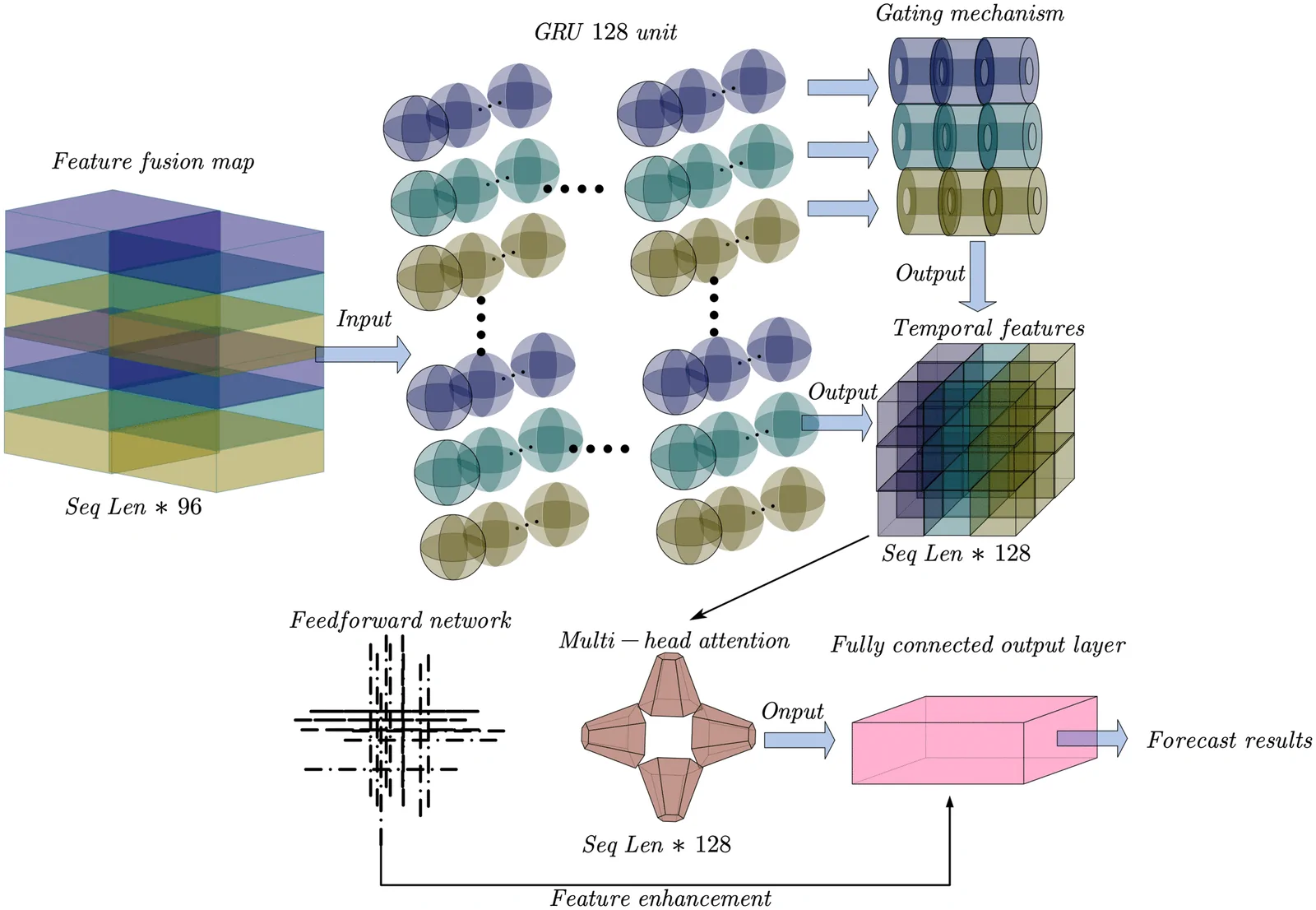
The GRU-Transformer collaborative mechanism. The GRU layer first captures medium-term evolution patterns (e.g., continuous changes in fuel moisture), retaining sensitivity to short-term disturbances; then the Transformer encoder mines cross‑time‑step contextual dependencies from a global perspective (e.g., the remote modulation of climate anomalies on fire risk). This progressive “local→global” extraction enables the model to respond to both rapid fuel variations and slow climate trends within a physically consistent framework, avoiding the long‑term forgetting of a standalone RNN or the local over‑smoothing of a standalone Transformer.


The GRU-Transformer collaborative mechanism [54] (Fig 5) uses a GRU layer to model temporal dependencies in multi-scale features, capturing mid-term evolution. The refined features are then processed by a Transformer encoder to mine global contextual correlations, and a fully connected layer generates the final fire risk prediction, enabling deep extraction from local to global insight. The mathematical expression is shown below.

$$\begin{array}{c} F=CNN\left(X_{\text{sho}rt},X_{mid},X_{long}\right) \end{array}$$ (21)

$$\begin{array}{c} H=GRU\left(F\right) \end{array}$$ (22)

$$\begin{array}{c} O=Transformer\left(H\right) \end{array}$$ (23)

$$\overset{⌢}{y}=FC\left(O\left[:,–1,\;:\right]\right)$$ (24)

where F denotes the fused multi-scale features, H represents the GRU output capturing temporal dependencies, O is the output of the Transformer encoder, and $\overset{⌢}{y}$ is the final FWI prediction, FC denotes the fully connected layer.

The core hyperparameter configuration of the GRU‑Transformer collaborative module (Table 5) includes parameters such as input dimensions, the output of the multi‑scale CNN, GRU layer, Transformer encoder, and output layer.

The input dimension of 96 (32 dimensions from each of the three CNN channels) ensures lossless fusion of multi-scale features; both the GRU hidden layer and the Transformer d_model are set to 128 to facilitate collaboration; nhead = 8 and the feed-forward network dimension of 512 (4 times d_model) follow the standard Transformer configuration. The number of GRU layers is set to 1 to avoid overfitting in deep sequential networks; the number of Transformer layers is 2, which is sufficient to capture global context. The output layer reduces dimensions progressively (128, 64, 32, 1), compressing the fused features into a single-value FWI prediction. Overall, this configuration seamlessly connects the outputs of the physical grouping (Table 3) and multi-scale convolution (Table 4) to the temporal-global fusion module, without introducing an additional hyperparameter tuning burden.

## 3. Data processing and analysis

### 3.1. Data source and description

The Huitong Forest Ecological Station provides 18 years of continuous observations (2005–2022) across three core datasets (Table 6): climate [55], hydrological [56], and soil [57]. Climate data shows 96.51% completeness, soil data includes 82 variables with 66.04% completeness, and hydrological data contains 89 variables. These form the basis for fire risk and ecosystem coupling analysis.

**Table 6:** Data set information.

Data set	Number of variables	number of observations	data integrity
Soil element data	82	4,458	66.04%
Climate and environment data	22	216	96.51%
Moisture element data	89	168,962	60.11%

Climate and environmental data have the highest completeness (96.51%), while soil and moisture data reach only 66.04% and 60.11%, respectively. This discrepancy stems from practical constraints in long‑term field observations: climate variables (temperature, humidity, etc.) are largely collected through automated systems, whereas soil and moisture data rely on manual or semi‑automatic measurements, resulting in more missing values. Within the fuel‑climate coupling framework, FWI and its sub‑indices (FFMC, DMC, DC) are highly dependent on the continuity of climate data; the high completeness of climate data provides reliable inputs for the model. In contrast, the lower completeness of soil and moisture features may introduce noise or bias. This suggests that appropriate imputation strategies should be adopted, and sensitivity analyses of low‑completeness features are necessary.

### 3.2. Data preprocessing process

Data cleaning and integration merged three datasets using year, month, day and ecological station code or plot code as keys into a unified table. Outliers were handled with the MAD method and ecological prior knowledge. To avoid data leakage, missing values were filled using a training‑set‑only K‑nearest neighbor algorithm: the dataset was first split chronologically into training (80%) and test (20%) sets, then the algorithm (k = 5, Euclidean distance) was fitted on the training set and applied to impute missing values in both sets. Feature engineering involved daily calculation of FWI system components FFMC, DMC, DC, ISI, BUI and FWI, as well as construction of temporal derivative features like consecutive dry days. From the initial 199 features, 21 were selected based on Pearson correlation with FWI (threshold |r| ≥ 0.6). No dimensionality reduction (e.g., PCA) was used, preserving physical interpretability. Data standardization performed Z-score normalization on all numerical features.


$$\begin{array}{c} X=\frac{X-\mu }{\sigma } \end{array}$$ (25)

Where μ is the mean of the feature and σ is the standard deviation.

### 3.3. Feature analysis and dataset construction

**Fig 6:**
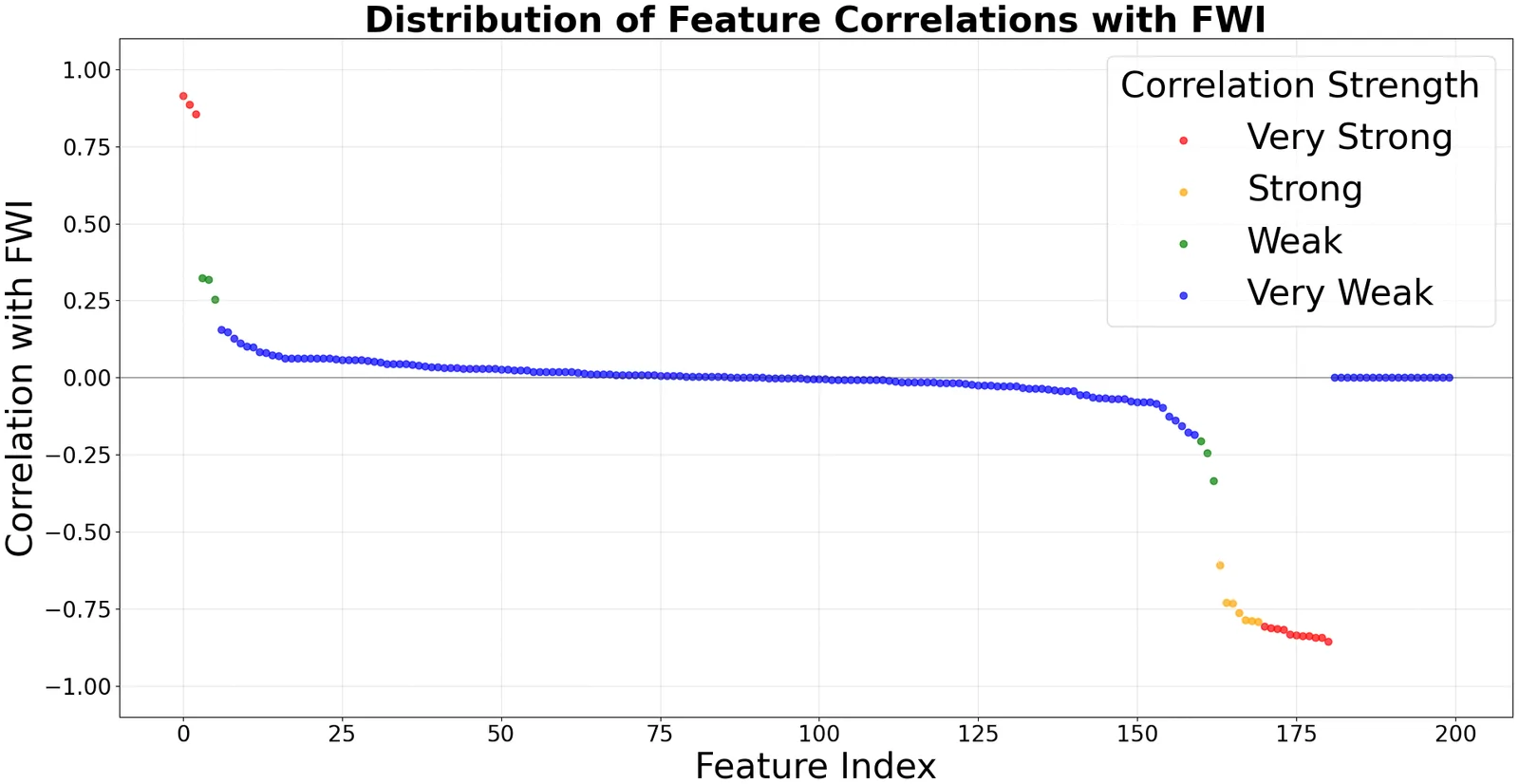
Distribution of feature correlations with FWI. The vast majority of features (173 out of 199) show weak correlations, while strong correlations are concentrated among a few temperature-related indicators and are predominantly negative (the monthly land surface temperature correlation coefficient reaches −0.855). This phenomenon indicates that, within the fuel‑climate coupling framework, FWI is not a linear superposition of multiple factors but is strongly modulated by key climatic factors such as temperature. The dominance of weakly correlated features also reflects that a single linear correlation is insufficient to fully characterize fire risk, necessitating a deep learning model to capture nonlinear interactions. This provides a data-driven justification for the subsequent multi-scale, physically aligned architecture design.

The three datasets contain 199 features. Correlation analysis (Fig 6) with FWI shows a concentrated distribution: 173 features have weak correlation between −0.1 and 0.1; three show very strong positive correlation between 0.8 and 1; 11 show very strong negative correlation between −0.8 and −1; and seven show strong negative correlation between −0.6 and −0.8. Negative correlations dominate, with monthly surface temperature showing the strongest correlation at −0.8550. Temperature-related indicators show strong negative correlation with FWI, highlighting the important role of temperature.

This scatter plot shows the distribution of feature correlations, with many features densely distributed near zero, indicating weak correlations, while a few extremely strong correlations (Table 7) appear at both ends. This visualization confirms the central tendency in statistics and highlights rare but impactful key features, providing an intuitive basis for dataset construction.

**Table 7:** Correlation coefficient.

Characteristic	Relevance with FWI	Physical driver grouping	Correlation intensity
ISI	0.91463	Climate driven group	Extremely relevant
FFMC	0.88486	Climate driven group	Extremely relevant
Monthly scale_air pressure (hPa)	0.85622	Soil driven group	Extremely relevant
Monthly Scale_UVIRradiation (MJ/m²)	−0.60941	Climate driven group	Strongly related
Monthly scale_photosynthethes effective radiation (MJ/m²)	−0.72987	Climate driven group	Strongly related
Tree trunk runoff_tree trunk runoff	−0.73082	Hydrological driven Group	Strongly related
BUI	−0.76349	Hydrological driven Group	Strongly related
Monthly scale_net radiation (MJ/m²)	−0.78567	Climate driven group	Strongly related
Monthly scale_60 cm soil temperature (°C)	−0.78809	Soil driven group	Strongly related
DMC	−0.79239	Hydrological driven Group	Strongly related
Monthly Scale_Reflected Radiation (MJ/m²)	−0.80724	Climate driven group	Extremely relevant
Monthly Scale_Total Radiation (MJ/m²)	−0.81088	Climate driven group	Extremely relevant
DC	−0.81479	Soil driven group	Extremely relevant
Temperature change rate	−0.81699	Climate driven group	Extremely relevant
Rainwater quality_sulfate root	−0.83240	Hydrological driven Group	Extremely relevant
Monthly scale_5 cm soil temperature(°C)	−0.83607	Soil driven group	Extremely relevant
Monthly scale_10 cm soil temperature (°C)	−0.83736	Soil driven group	Extremely relevant
Evaporation amount_water storage capacity of the soil layer on that day	−0.83800	Hydrological driven Group	Extremely relevant
Monthly scale_temperature (°C)	−0.84368	Soil driven group	Extremely relevant
Temperature anomaly	−0.84873	Climate driven group	Extremely relevant
Monthly scale_surface temperature(°C)	−0.85499	Climate driven group	Extremely relevant

Among the 21 strongly correlated features, positive correlations only appear for ISI, FFMC, and monthly mean air pressure; the remaining features show negative correlations, with radiation, soil temperature, DMC, DC, and other indicators exhibiting strong negative correlations with FWI (absolute values all > 0.73). This is consistent with the physical grouping in Table 3: ISI and FFMC belong to the short-term climate-driven group, reflecting the positive contribution of wind speed and surface fuel moisture to fire risk; whereas soil temperature (5 cm, 10 cm, 60 cm), DC, and others belong to the long-term soil-driven group, and their negative correlation suggests that soil heat accumulation is associated with lower fire risk. This correlation distribution provides a statistical basis for the physical mapping of the kernel sizes in Table 4 (7 days for short-term, 30 days for medium-term, and 90 days for long-term).

Due to the differences in the sizes of the three feature-grouped datasets, the three datasets were uniformly adjusted to 216 monthly records. To meet the model’s requirement for high temporal resolution, the monthly data were expanded to 6,570 daily records through linear interpolation. On this basis, a sliding window (window length of 180 days, step size of 1 day) was applied to generate 6,390 base sequence samples, and multi-scale sampling with window lengths of 90, 120, 150, 210, and 240 days was introduced, padded to 180 days. Gaussian noise augmentation with standard deviations of 0.01 and 0.02 was added, ultimately yielding 33,560 samples for model training and evaluation. For rigorous evaluation, all sequence samples generated in chronological order were directly split into the first 80% for training and the last 20% for testing.

## 4. Experiment and proof

To comprehensively evaluate the performance of the proposed FWI-MSNet model in forest fire risk prediction tasks, we designed and conducted a series of rigorous experiments.

### 4.1. Evaluation metrics

This study used four widely recognized indicators, RMSE, MAE, R^2^, and MAPE, to quantitatively evaluate the performance of the regression prediction task. The mathematical expressions are as follows:


$$\begin{array}{c} RMSE=\sqrt{\frac{\text{1}}{N}\sum_{\text{i}=1}^{N}(y_{i}-{\hat{y}}_{i}{)}^{\text{2}}} \end{array}$$ (26)

$$\begin{array}{c} MAE=\frac{\text{1}}{N}\sum_{i=1}^{N}\left|y_{i}-{\hat{y}}_{i}\right| \end{array}$$ (27)

$$\begin{array}{c} R^{\text{2}}=1-\frac{\sum_{i=1}^{N}{\left(y_{i}-{\hat{y}}_{i}\right)}^{\text{2}}}{\sum_{i=1}^{N}{\left(y_{i}-\overset{―}{y}\right)}^{\text{2}}} \end{array}$$ (28)

$$\begin{array}{c} MAPE=\frac{100\%}{N}\sum_{i=1}^{N}\left|\frac{y_{i}-{\hat{y}}_{i}}{y_{i}}\right| \end{array}$$ (29)

Among them, N is the total number of samples; $y_{i}$ is the true value of the i sample; ${\hat{y}}_{i}$ is the predicted value of the i sample; and $\overset{―}{y}$ is the evaluation value of the real value.

### 4.2. Model fitting analysis

**Fig 7:**
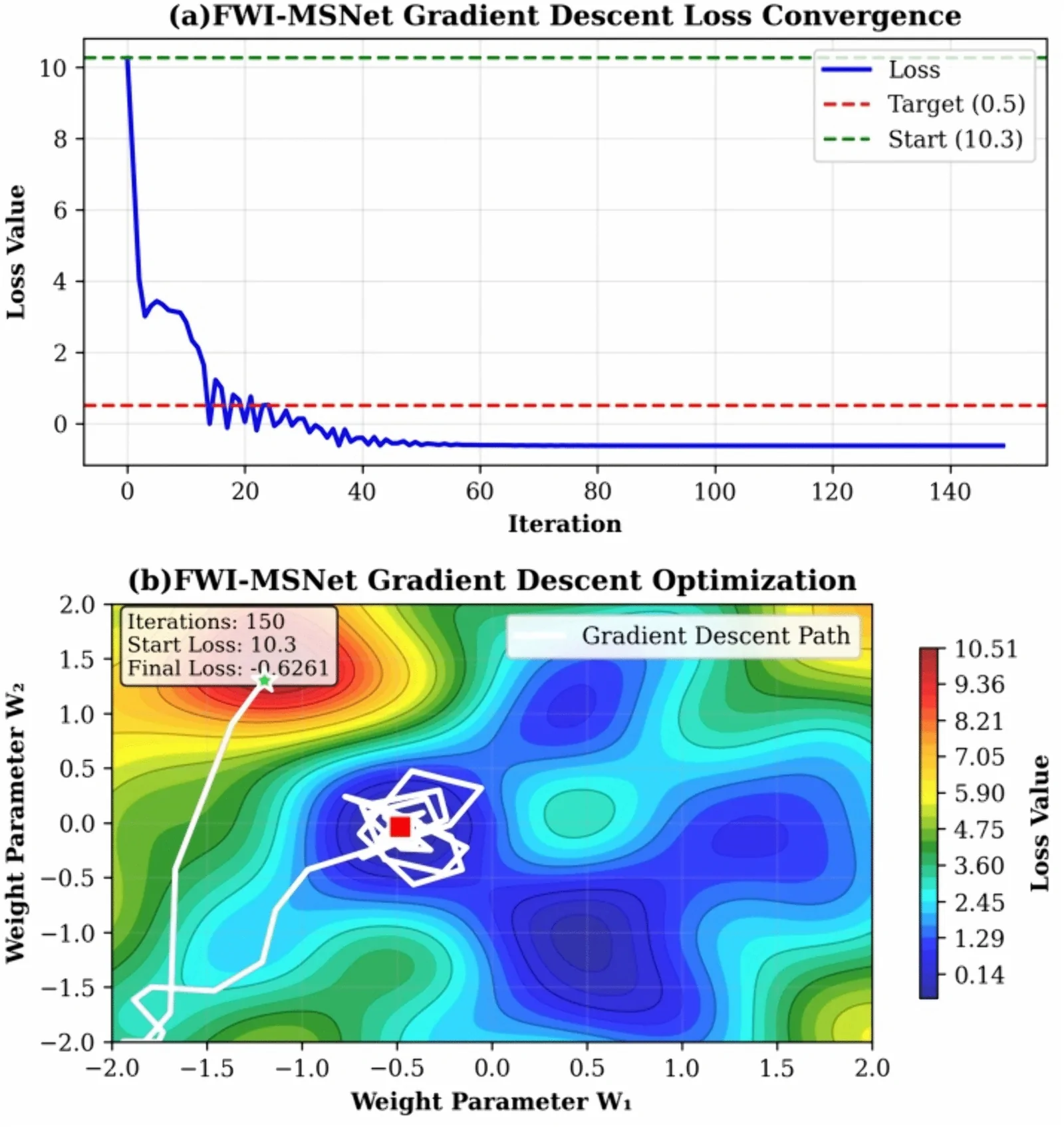
The fitting process of the FWI-MSNet model. The loss curve (Fig 7a) shows that the initial loss of 10.3 decreases rapidly within 20 epochs, then enters a phase of slight fluctuations and convergence, indicating that the model can quickly capture the dominant gradient direction of the fuel‑climate coupling. After 150 epochs, the loss stabilizes at −0.626 with no obvious overfitting. In the parameter contour map (Fig 7b), W_1_ and W_2_ migrate smoothly from a high‑loss region to a global low‑loss region along the gradient descent path, consistent with the loss curve. This convergence behavior demonstrates that the collaborative structure of multi‑scale convolution and GRU‑Transformer does not increase optimization difficulty; instead, it provides a clear gradient descent path through physically aligned feature extraction.

The fitting process of the FWI-MSNet model is illustrated (Fig 7). The loss convergence curve (Fig 7a) shows that the initial loss of 10.3 decreases rapidly, enters a fluctuating convergence phase after about 20 rounds, and reaches −0.626 after 150 iterations. A contour map of parameter optimization (Fig 7b) shows weight parameters W_1_ and W_2_ migrating from a high‑loss area to a low‑loss area along the gradient descent path, with a final loss consistent with (Fig 7a). Together, they indicate fast and successful convergence and effective parameter updates via gradient descent in FWI-MSNet.

### 4.3. Comparative experiments

To verify the effectiveness of each module in FWI-MSNet, this study designed two types of comparative experiments: ablation experiments and traditional model comparisons. All models predict the next 30 days. The ablation models include models without multi-scale convolution, without Transformer, without GRU, and without FWI features, all based on FWI-MSNet. The traditional models include XGBoost, LSTM, and CNN.

All models in this study were trained and evaluated under identical conditions to ensure fair comparison. Specifically, the same data split (chronological 80/20), feature normalization, batch size of 32, optimizer (Adam, initial learning rate 0.001), loss function (MSE), early stopping patience (20 epochs), and maximum epochs (200) were used for every deep learning model (including FWI-MSNet, its ablation variants, LSTM, and CNN). XGBoost was trained with grid-searched hyperparameters (number of trees = 100, max depth = 6, learning rate = 0.1).

**Fig 8:**
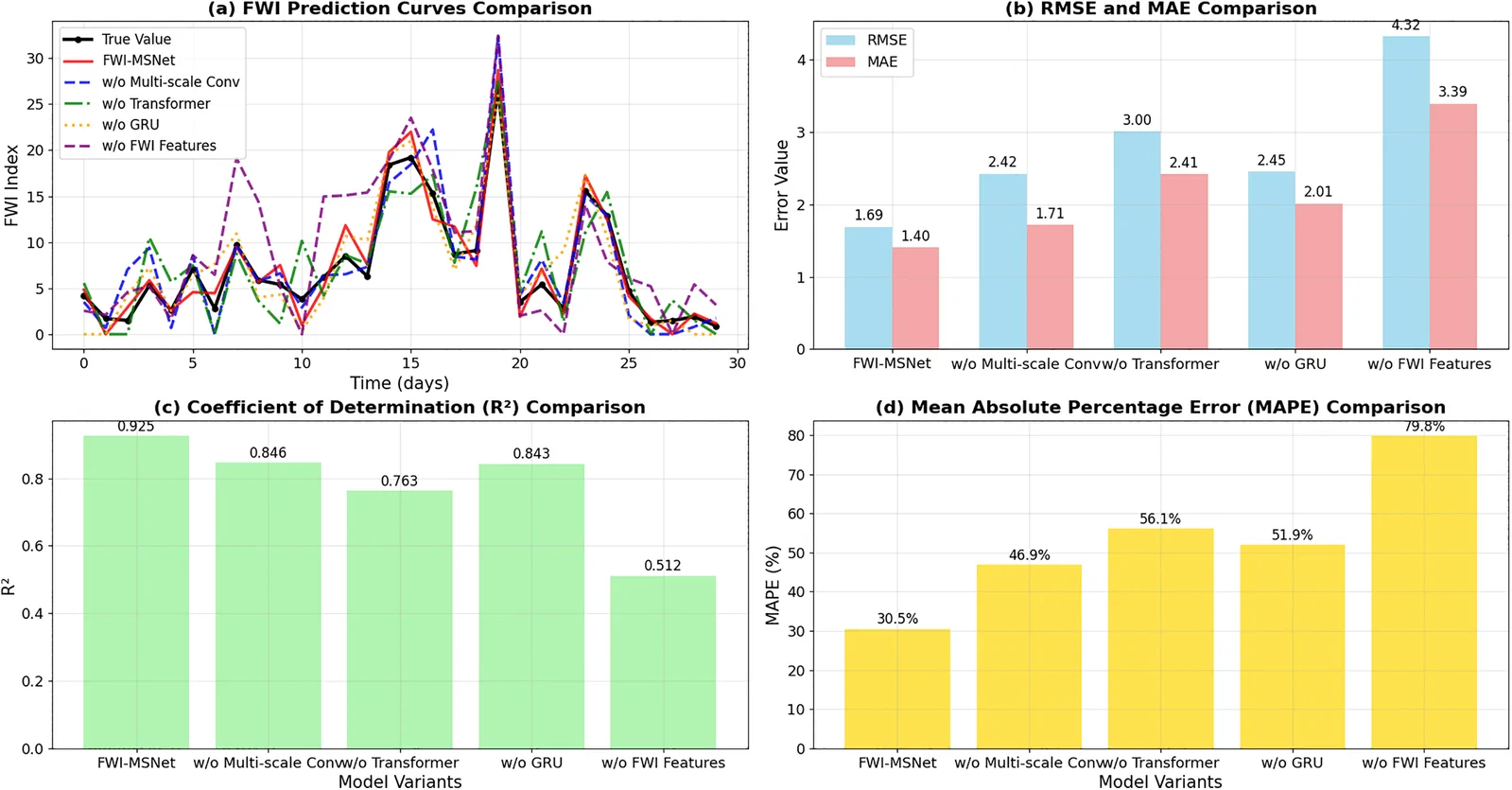
The ablation experiment. FWI‑MSNet outperforms all variants in both RMSE (1.48) and MAE (1.10), while the model using only FWI features (without multi‑scale convolution and GRU‑Transformer) has an R^2^ as low as 0.512. This gap indicates that relying solely on the FWI index itself is insufficient to capture the nonlinear fuel‑climate coupling; multi‑scale feature extraction and temporal‑global fusion mechanisms are essential. Most variants achieve stable R^2^ values between 0.83 and 0.85, but the MAPE ranges widely (31.6%–76.1%), reflecting differences in prediction sensitivity to extreme fire risk values across architectures. Overall, these results validate the critical role of a physically aligned framework in improving prediction accuracy and robustness.

The ablation experiment (Fig 8) shows model variants closely follow true FWI trends. Error metrics (Fig 8b) reveal significant differences: the Hybrid model achieves the lowest RMSE of 1.48 and MAE of 1.10. Most models (Fig 8c) show good fit with R^2^ values between 0.83 and 0.85, while the FWI Features model is lower at 0.512. MAPE comparisons (Fig 8d) range from 31.6% to 76.1%. Overall, these results demonstrate the superior performance of FWI-MSNet in FWI prediction.

**Fig 9:**
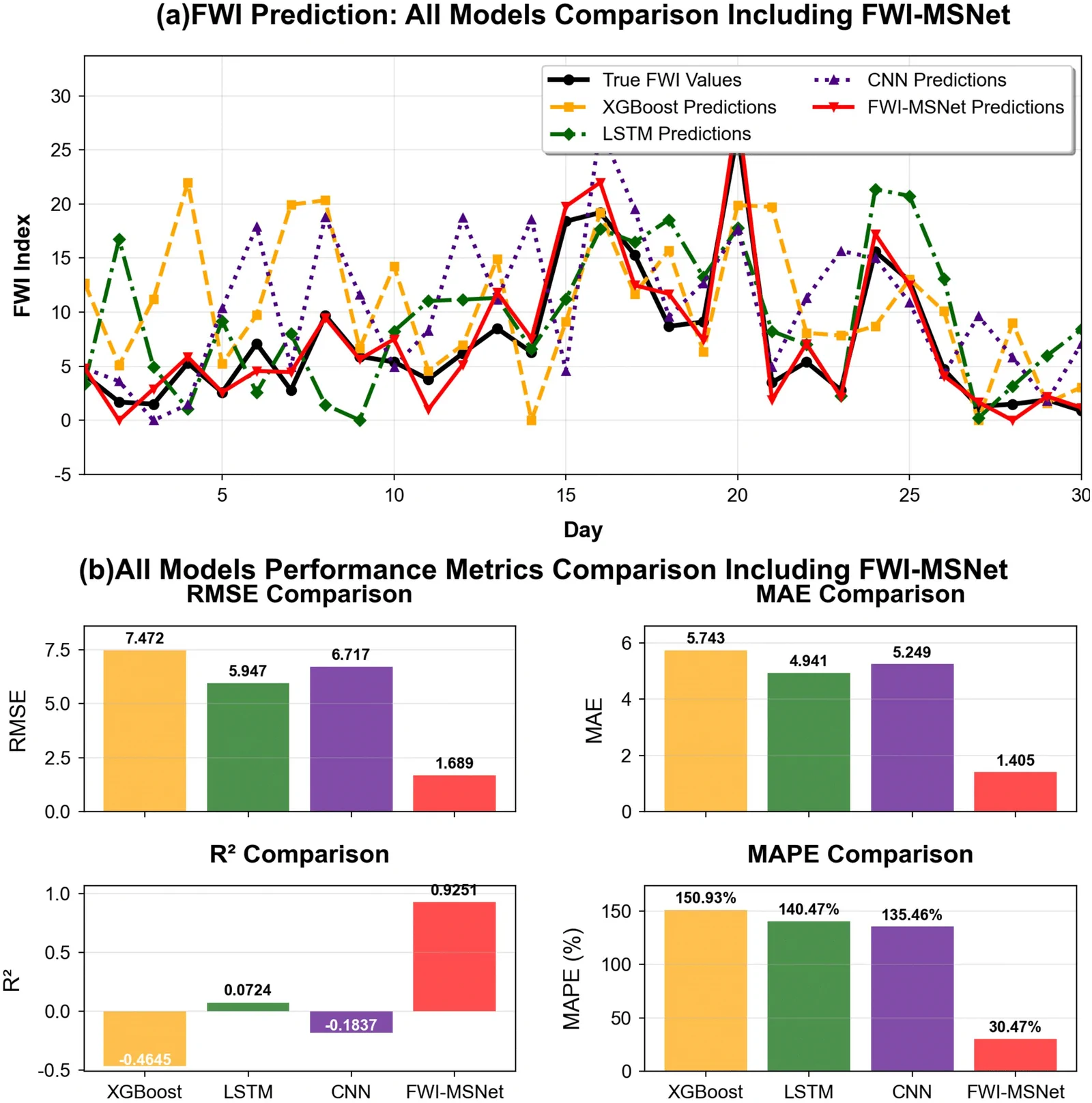
The traditional model comparison. Compared with XGBoost, LSTM, and CNN, FWI‑MSNet achieves the highest agreement between its predicted curve and the true FWI values, with RMSE (1.889), MAE (1.405), and MAPE (30.47%) all significantly better than those of the other models, and an R^2^ of 0.9251. This advantage stems from the physically aligned design of the framework: although traditional models such as LSTM can capture temporal dependencies, they lack explicit multi‑scale convolution modeling of fuel moisture variations from daily to seasonal scales; XGBoost struggles with long‑range climate–fuel coupling. By leveraging parallel multi‑scale 1D‑CNN and GRU‑Transformer collaboration, FWI‑MSNet responds simultaneously to rapid fuel changes and slow climate trends, thus achieving superior performance in both extreme value fitting and trend tracking.

The traditional model comparison (Fig 9) shows that FWI-MSNet’s red prediction curve fits the true FWI values significantly better than CNN, XGBoost, and LSTM models (Fig 9a). FWI-MSNet achieves superior performance (Fig 9b) with an RMSE of 1.889, MAE of 1.405, R^2^ of 0.9251, and MAPE of 30.47%, far exceeding other models.

On the training set (Table 8), FWI-MSNet achieves the best performance (RMSE = 1.52, R^2^ = 0.94, MAPE = 28.2%). Removing any key component (multi-scale convolution, Transformer, GRU, or FWI features) significantly degrades all metrics, with the exclusion of FWI features causing the worst drop (R^2^ = 0.56, MAPE = 73.4%). Conventional models (XGBoost, LSTM, CNN) perform poorly, with XGBoost yielding a negative R^2^. These results confirm the effectiveness and necessity of each module in FWI-MSNet.

**Table 8:** The results of the training set.

Models	RMSE	MAE	R^2^	MAPE(%)
FWI-MSNet	1.5213	1.2831	0.9412	28.15%
Without multi-scale convolution	2.1804	1.5413	0.8614	43.12%
Without Transformer	2.7042	2.1727	0.7869	51.58%
Without GRU	2.2019	1.8099	0.8583	47.79%
Without FWI features	3.8839	3.0475	0.5562	73.38%
XGBoost	6.7247	5.1688	−0.3181	138.86%
LSTM	5.3519	4.4466	0.1589	129.23%
CNN	6.0457	4.7237	−0.0653	124.62%

Removing multi‑scale convolution increases RMSE to 2.18, indicating that a single scale cannot simultaneously capture the dual response of fuel moisture to meteorological disturbances (weekly) and drought accumulation (seasonal). After removing the Transformer, R^2^ drops to 0.787, suggesting that global contextual dependencies (e.g., remote modulation by climate anomalies) are not effectively modeled. Removing GRU results in an MAE of 1.81, reflecting the loss of medium‑term temporal evolution information. The most severe performance degradation occurs when FWI features are removed (R^2^ = 0.556), confirming that the core fire risk index serves as a physical anchor for fuel‑climate coupling. Traditional models (XGBoost, LSTM, CNN) all achieve R^2^ values below 0.16 or even negative, due to their lack of physically aligned multi‑scale and collaborative mechanisms.

On the test set (Table 9), against seven models including no multi-scale convolution and XGBoost, FWI-MSNet reduced RMSE by 55.7%, MAE by 52.7%, and MAPE by 60.0%. Compared with each model, the arithmetic mean of RMSE, MAE, and MAPE improved by 56.1%, and the average absolute increase in R^2^ was 0.584, reflecting its significant superiority in prediction accuracy.

**Table 9:** Comparative experimental results.

Models	RMSE	MAE	R^2^	MAPE(%)
FWI-MSNet	1.6892	1.4046	0.9251	30.47%
Without multi-scale convolution	2.4227	1.7126	0.8460	46.88%
Without Transformer	3.0047	2.4141	0.7632	56.07%
Without GRU	2.4466	2.011	0.8430	51.94%
Without FWI features	4.3154	3.3861	0.5115	79.76%
XGBoost	7.4719	5.7431	−0.4645	150.93%
LSTM	5.9466	4.9407	0.0724	140.47%
CNN	6.7174	5.2485	−0.1837	135.46%

The comparative experimental results further validate the generalization advantage of FWI‑MSNet on the test set. Compared with the training set (Table 8), all models show increased errors, but FWI‑MSNet still maintains an RMSE of 1.69 and an R^2^ of 0.925, with the smallest degradation, indicating that the physically aligned design effectively suppresses overfitting. Removing FWI features causes R^2^ to drop to 0.512, the largest decline, highlighting the anchoring role of the core fire risk index in cross‑sample prediction. Removing the Transformer or the GRU increases RMSE to 3.00 and 2.45, respectively, demonstrating that temporal‑global collaboration is particularly critical for unknown fluctuations on the test set. Traditional models (XGBoost, CNN) still yield negative R^2^ values, suggesting that pure data‑driven methods lacking physical priors struggle to generalize.

Based on the evaluation results on the training and test sets, FWI-MSNet achieves an R^2^ of 0.9412 on the training set and 0.9251 on the test set, with RMSE only slightly increasing from 1.52 to 1.69 and MAPE marginally rising from 28.2% to 30.5%. No significant degradation is observed in any metric on the test set, and the performance trends of the ablation models and baseline models remain consistent. This indicates that FWI-MSNet has good fitting capability while effectively avoiding overfitting, demonstrating robust generalization performance.

**Fig 10:**
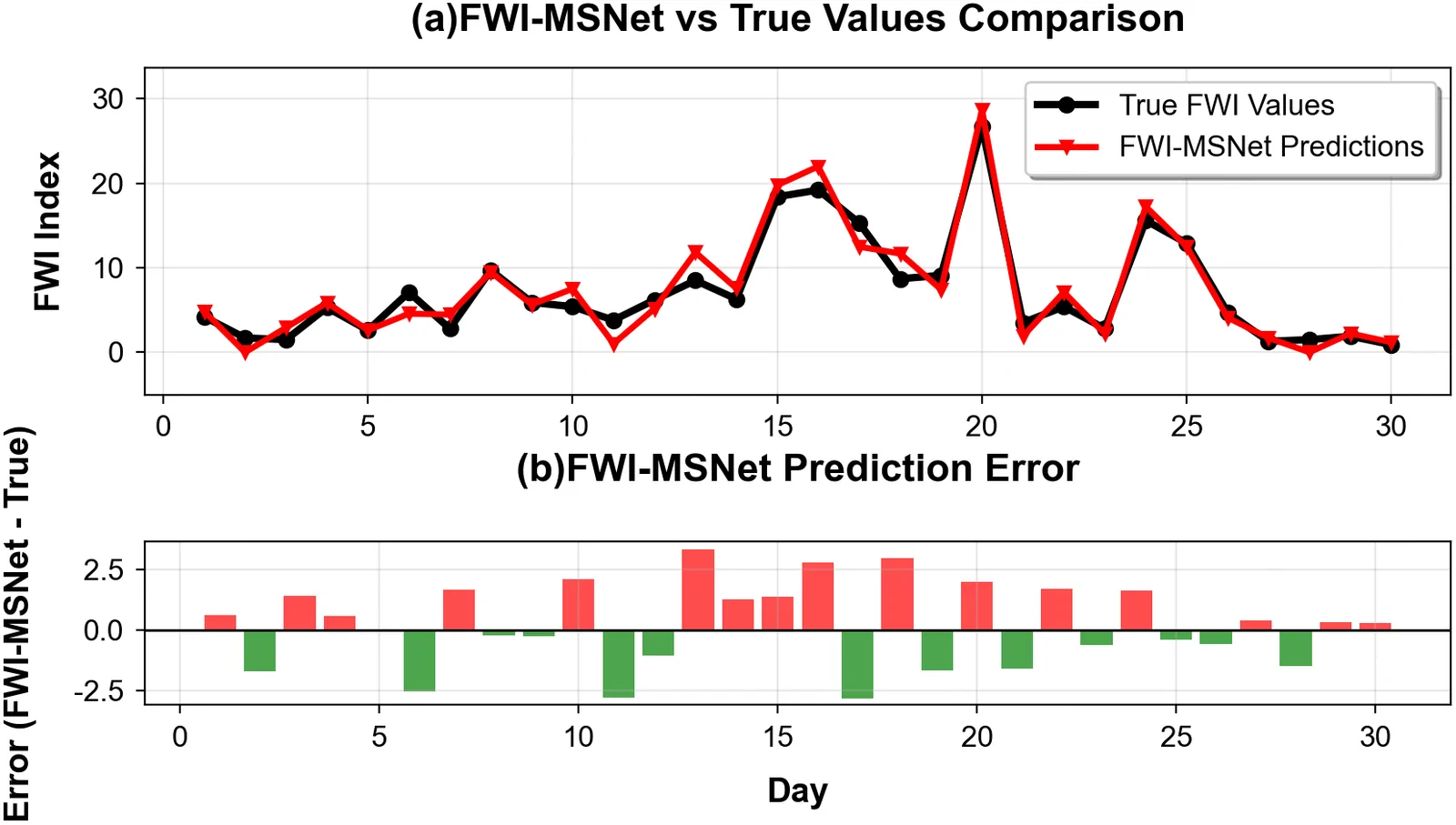
FWI-MSNet prediction results. The prediction error remains within ±2.5 at all times. Even when the true FWI values exhibit significant fluctuations (e.g., days 15–25), the model responds promptly without lag. This indicates that the multi-scale features (daily to seasonal) extracted by the framework, together with the GRU-Transformer collaborative mechanism, effectively capture the rapid response and slow recovery of fuel moisture to climate anomalies. The stable error range also suggests that the model has the potential to provide reliable early warnings in real-world fire prevention scenarios.

The predicted curve (Fig 10) closely matches true FWI values and captures dynamic risk changes even during significant fluctuations, while prediction errors remain within ±2.5, demonstrating stable performance with practical value for forest fire prevention.

### 4.4. Case study experiment

Australia’s catastrophic 2020 forest fires were triggered by lightning and human activities under extreme drought and windy conditions, burning 18.6 million hectares. To test FWI-MSNet’s robustness, this study used ERA5 data [58] and six representative areas covering diverse ecosystems. The FWI values for the case study are calculated using the same mathematical formulations described in Section 2.1, driven by ERA5 meteorological inputs (temperature, relative humidity, wind speed, and precipitation).

**Fig 11:**
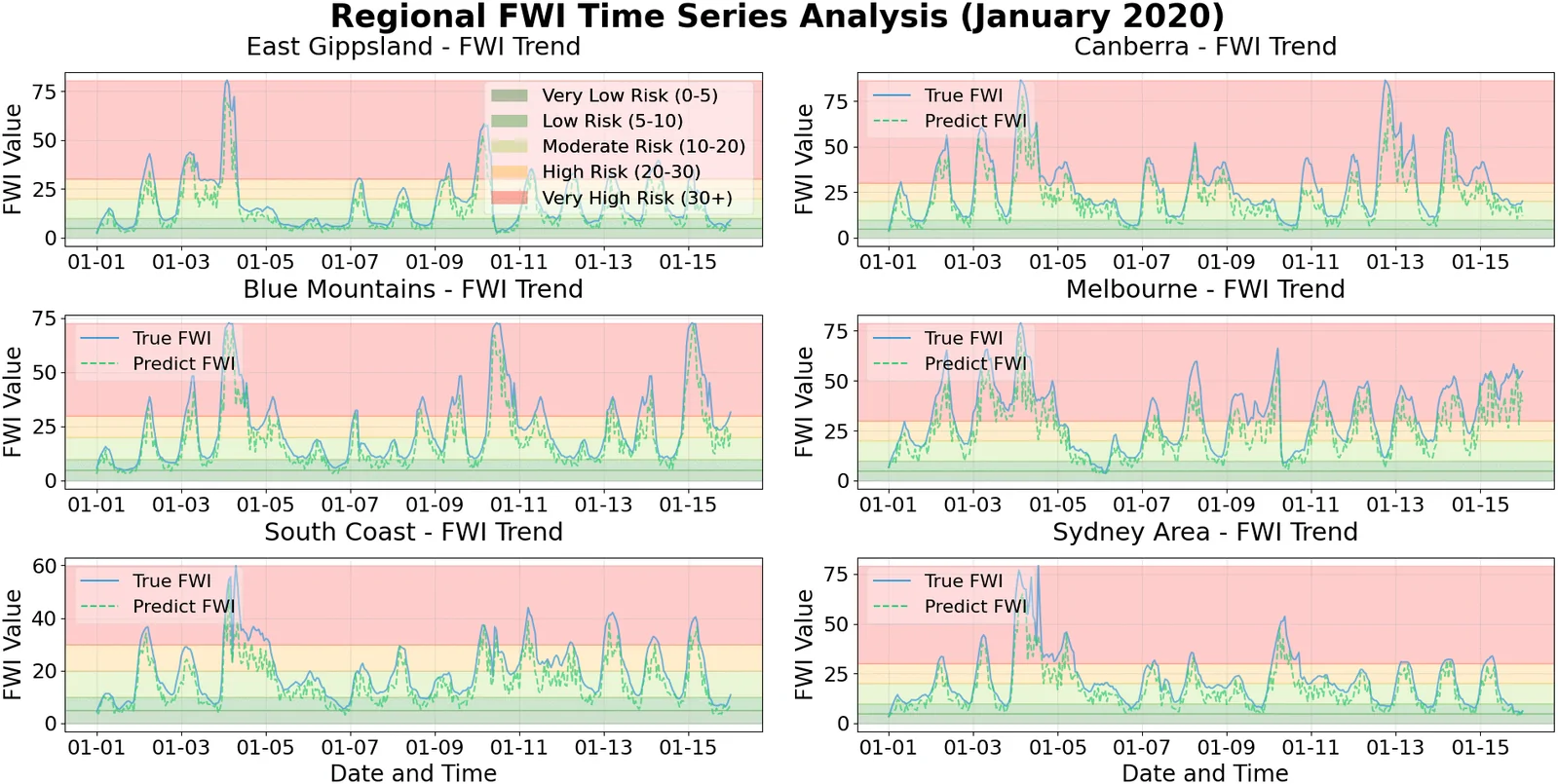
The FWI predictions for forest fires in Australia. The model demonstrates cross-regional generalization capability in six regions of Australia. The model performs better in forest-dominated areas (e.g., East Gippsland, R^2^ > 0.76) than in urban areas (e.g., Melbourne), which is related to the regional consistency of the fuel‑climate coupling mechanism: forest areas have continuous fuel loads and moisture response patterns more similar to the training domain, whereas the surface conditions and anthropogenic disturbances in urban areas reduce the applicability of physical alignment. The MAPE remains stable at around 25%, and there is no significant lag in risk level transitions, indicating that the multi‑scale feature extraction has learned universal fire evolution patterns across ecological regions rather than over‑relying on local statistical characteristics.

The FWI predictions for forest fires in Australia (Fig 11) show that the FWI-MSNet model effectively captures the evolution of fire risk—from low to extreme—across the six regions. During risk-level transitions, the predicted curve aligns with the actual trend, without significant lag. Quantitative evaluation further demonstrates the model’s robust cross-regional generalization: it achieves R^2^ values ranging from 0.57 to 0.77 and a stable MAPE of approximately 25% across all regions, despite being trained solely on data from Huitong, China. Notably, the model performs best in forest-dominated regions such as East Gippsland and the Blue Mountains, where R^2^ exceeds 0.76 (Table 10), while showing relatively lower performance in urban areas like Melbourne—a pattern consistent with the ecological similarity to the training domain. This case study confirms the model’s capability to accurately track fire risk dynamics with reliable transferability across regions, offering a valuable reference for forest fire prevention decision-making.

**Table 10:** The evaluation results.

Region	RMSE	MAE	R^2^	MAPE
East Gippsland	6.7502	4.7096	0.7678	25.5119
Canberra	9.7585	7.194	0.6744	25.1229
Blue Mountains	8.0932	5.9905	0.7618	25.3189
Melbourne	10.3667	8.0135	0.5676	24.5804
South Coast	6.5509	5.0417	0.6151	25.0579
Sydney Area	7.2271	5.368	0.6999	24.489

Model performance in forest-dominated areas (East Gippsland, Blue Mountains) yields R^2^ values above 0.76, while in the urban area (Melbourne) it drops to 0.57. This discrepancy may be attributed to the forest cover characteristics of the training domain (Huitong Ecological Station, China): forest areas have high fuel continuity and moisture response patterns similar to Huitong, making the multi-scale coupling laws learned by the model more transferable; urban areas, however, are subject to disturbances from artificial underlying surfaces and fire suppression interventions, which weaken the effectiveness of physical alignment. The MAPE remains stable at around 25% across all regions, with RMSE ranging between 6.55 and 10.37, indicating that the model’s tracking of extreme fire danger level transitions is consistent, although attention should be paid to the impact of data scale (FWI value range) on error evaluation.

### 4.5. Insights from deep learning compared to alternative models

The novelty of this framework lies not in proposing a new deep learning architecture, but in establishing a physically aligned framework that explicitly couples the FWI system’s multi-scale structure with the model design. Beyond prediction accuracy, FWI-MSNet provides scientific insights that traditional models (XGBoost, LSTM, CNN) cannot offer. Based on the quantitative results reported in Table 9, three key findings emerge. First, the FWI system serves as a critical physical anchor for fire risk prediction: removing FWI features degrades R^2^ from 0.9251 to 0.5115, a 44.7% decrease relative to the full model. This confirms that the FWI system is not merely an additional input variable but a foundational physical prior that guides the learning process—a finding that traditional models, which treat all inputs uniformly, cannot reveal. Second, the simultaneous modeling of daily, monthly, and seasonal scales is essential: removing multi-scale convolution increases RMSE from 1.6892 to 2.4227, a 43.4% increase. This demonstrates that the fuel-climate coupling mechanism operates across multiple temporal scales simultaneously, and a single-scale model (e.g., standard CNN or LSTM) inevitably misses critical information at other scales. Third, the GRU and Transformer modules play distinct but complementary roles: removing GRU increases RMSE to 2.4466, while removing Transformer increases it to 3.0047, indicating that GRU primarily captures local sequential evolution of fuel moisture, whereas Transformer captures global cross-period correlations (e.g., remote modulation by climate anomalies). These insights, derived directly from the ablation experiments in Table 9, demonstrate that the physically aligned deep learning framework not only improves prediction accuracy but also provides interpretable understanding of the multi-scale fuel-climate coupling mechanism—a capability that conventional black-box models lack.

## 5. Discussion

Despite the promising results, this study has three main limitations. First, the model is trained exclusively on data from Huitong Ecological Station in China. Although its generalizability was tested on the 2020 Australian wildfire case study, validation across a broader range of geographic regions and climatic conditions remains necessary. Additionally, the current dataset lacks critical spatial information such as satellite-derived vegetation indices and topographic features (e.g., slope, aspect) that are known to influence fire behavior. Second, the current architecture operates on point-based time series without explicitly modeling spatial fire propagation across landscapes, and the GRU-Transformer mechanism introduces significant computational complexity. Third, although physically aligned in feature organization, the Transformer’s self-attention mechanism remains largely opaque, and the model does not output intermediate physical states (e.g., predicted moisture codes) that could be verified against physical equations, limiting full interpretability for domain experts.

Building on these limitations, future work will pursue three directions:

(1) incorporating remote sensing data (Landsat, MODIS) and topographic variables to enable grid-based regional risk assessment;
(2) exploring Graph Neural Networks (GNNs) to explicitly model fire spread across geographical grids while reducing computational costs via lightweight attention mechanisms;
(3) developing a more interpretable model with auxiliary tasks to predict intermediate FWI components and physical loss functions that enforce known relationships (e.g., water balance constraints). Addressing these challenges will be crucial for evolving FWI-MSNet into a more robust, generalizable, and interpretable operational forecasting system.

## 6. Conclusions

This study proposed FWI-MSNet, a physically aligned deep learning framework for forest fire risk prediction based on 18 years of synchronous observation data from Huitong Ecological Station in China. The main findings are summarized as follows:

(1) FWI-MSNet outperformed all seven baseline models, achieving an RMSE of 1.6892, MAE of 1.4046, R^2^ of 0.9251, and MAPE of 30.47%.
(2) Physical alignment of features according to the FWI system’s multi-scale temporal structure substantially improved prediction accuracy; removing FWI features degraded R^2^ to 0.5115.
(3) The model accurately captured the fire risk evolution trajectory in the 2020 Australian catastrophic wildfires, demonstrating potential generalizability beyond the training region.

### Declaration of Generative AI and AI-assisted technologies in the writing process

The authors confirm that there was no use of Artificial Intelligence (AI)-Assisted Technology for assisting in the writing or editing of the manuscript and no images were manipulated using AI.
