# Supplementary material for: Physically aligned forest fire risk prediction: A deep learning framework coupling fuel and climate multivariate factors
Source: PLoS One. 2026 Sep 1;21(9):e0355829. doi: 10.1371/journal.pone.0355829 (PMC13533387; doi:10.1371/journal.pone.0355829)
Supplement: Supplementary file 1 [file pone.0355829.s001.pdf]

Reviewer Report

Manuscript Title:
"Physics-Informed Forest Fire Risk Prediction: A Deep Learning Framework Coupling Fuel and Climate Multivariate Factors"

Recommendation

The topic is important and the pipeline is reasonable, but several key claims and evaluation details limit confidence. I recommend **major revision** to strengthen (a) the definition of the prediction task and inputs construction, (b) training leakage control, and (c) "physics-informed" claims in the title.

Major Comments

1. **"Physics-informed" is currently not demonstrated in the standard sense:** The manuscript positions FWI as "the physical core" and describes the framework "physics-informed". But the model appears to be a data-driven architecture guided by feature grouping rather than physics-informed optimization. The model does not explicitly incorporate physical constraints or differentiable governing equations. Please either rename the approach or add a true physics-informed component.
2. **High risk of leakage in evaluation:** The paper reports a random stratified split into train/val/test (70/15/15). For time series data this can introduce temporal leakage. Also, the model uses multi-scale convolutions with long-term path (up to a quarter), which could cause leakage. Please ensure that no overlapping windows cross split boundaries.
3. **Sample construction and dataset description are unclear:** The reported dataset size is inconsistent across sections. Also, please define at least one sample precisely, including the timestamp resolution, window length used to build features.
4. **Australia 2020 case study & Figure 10: clarify prediction vs ground truth:** As presented, Figure 10 reads more like a descriptive time-series plot than "prediction vs reference" evaluation. Is the "Hourly FWI" FWI-MSNet predictions or computed from ERA5?

Minor Comments

1) Section 4.1, "The Huitong Forest Ecological Station provides 18 years of continuous observations (2005-2012)…", which is 8 years. Please correct.

2) There are multiple grammar issues throughout. Please perform a careful English checking and improve clarity.
