# Supplementary material for: Physically aligned forest fire risk prediction: A deep learning framework coupling fuel and climate multivariate factors
Source: PLoS One. 2026 Sep 1;21(9):e0355829. doi: 10.1371/journal.pone.0355829 (PMC13533387; doi:10.1371/journal.pone.0355829)
Supplement: Supplementary file 2 [file pone.0355829.s003.docx]

**Response to Reviewers**

Dear Editor and Reviewers,

On behalf of all authors of this manuscript (Manuscript ID: PONE-D-26-00599), we sincerely thank the editorial team and the anonymous reviewers for their insightful comments and constructive suggestions, which have greatly improved the quality and clarity of the paper. We have carefully addressed each point in the revised manuscript. The comments fall into three categories: Key Revision Suggestions (10 questions), Reviewer #1 Report (6 questions), and Reviewer #2 Report (11 questions). Below is our point-by-point response. All revisions are marked in red. Should you have any further questions, please do not hesitate to contact us. We hope that the revised manuscript now meets the publication standards of your journal.

**Key Revision Suggestions**

**1. The manuscript proposes a deep learning framework; however, the source code and access to this framework are not provided. Please provide the source code of the developed framework for journal readers. You may upload it to a repository or upload center and provide its link in the methodology section.**

***Author’s Reply:***

Thank you for this important suggestion. We fully agree that sharing source code is essential for reproducibility. Accordingly, we have added the GitHub link (https://github.com/CBJYB/FWIMSNet-Forest-Fire-Risk-Prediction-Framework) to the framework source code in Section 2.5 (Methodology) on page 11. The repository includes a complete README and demo scripts to assist readers in using the framework.

2. **Restructure the methodology based on the provided source code to present a step-by-step guideline for journal readers showing how to use this framework.**

***Author’s Reply:***

Thank you for this constructive suggestion. In the revised manuscript, we have restructured the methodology section and added a step-by-step guideline in Section 2.5 (Methodology, starting on page 11), following the workflow of the source code. The overall execution flow includes data preprocessing (standardization and data augmentation), multi-scale input feature construction, model construction (multi-scale feature extraction, GRU-Transformer collaborative mechanism, and output layer), model training and evaluation, and finally prediction and evaluation. These steps are described in detail to help readers easily understand and use the proposed framework.

3. **Sections 2 and 3 can be merged into a single section titled Methodology.**

***Author’s Reply:***

Thank you for this helpful suggestion. In the revised manuscript, we have merged the original Sections 2 and 3 into a single Methodology section (now Section 2, beginning on page 6) and updated the section title accordingly. The subsections have also been renumbered for clarity.

4. **The Introduction requires substantial revision in terms of both quality and quantity. Please include a more comprehensive literature review, clearly define the research gaps, and then highlight the novelty of the current study.**

***Author’s Reply:***

Thank you for this constructive suggestion. We have substantially revised the Introduction (Section 1, starting from page 2) to address the concerns regarding both quality and quantity. Specifically:

More comprehensive literature review: We have systematically reviewed traditional sensor‑based methods, machine learning approaches, deep learning models, and physically‑enhanced methods. The revised Introduction now cites up‑to‑date works (references [5]–[16]) covering WSNs, random forest, gradient boosting, SVM, CNN‑BiLSTM, LSTNet, Transformer, enhanced FWI, and GIS‑based risk mapping.

Clearly defined research gaps: After the literature review, we explicitly point out three critical gaps: (1) most deep learning models treat fire prediction as a purely data‑driven task, overlooking the inherent multi‑scale temporal structure of fire physics; (2) the FWI system’s potential as a physically aligned organizational principle for feature engineering remains largely untapped; (3) existing architectures struggle to capture multi‑scale temporal patterns (daily to seasonal) and long‑term dependencies simultaneously.

Highlighted novelty: In response to the identified gaps, we list the three main contributions of our study: physically aligned feature organization, multi‑scale feature extraction, and temporal‑global fusion (GRU‑Transformer).

In addition, we have added a new Figure 1 with a data‑driven analysis of key fire indicators from the China Statistical Yearbook (2000–2023) to strengthen the motivation.

New references added in the revised Introduction (compared to the original version):

[5] (WSN real‑time monitoring), [6] (sensor types), [7] (early alarm), [8] (WSN challenges), [9] (Khurram et al., random forest & GBM), [10] (Chen et al., SVM), [11] (Škurić Kuraži et al., soil‑based index), [12] (Marjani et al., CNN‑BiLSTM), [13] (Lin et al., LSTNet), [14] (Miao et al., window‑enhanced Transformer), [15] (Santos et al., enhanced FWI), [16] (Kumari et al., GIS‑based risk).

We are grateful for the reviewer’s insightful suggestions, which have significantly improved the comprehensiveness and depth of our Introduction.

**5. The methodology section (currently Sections 2 and 3) needs better organization. Please restructure it based on the newly added source code. These sections also require more supporting references. In addition, each subsection should specify which packages or libraries were used.**

***Author’s Reply:***

Thank you for these valuable suggestions. In the revised manuscript, we have thoroughly restructured the methodology section based on the workflow of the newly added source code. First, we merged the original Sections 2 and 3 into a single Section 2 (Methodology) starting from page 6, following the logical flow: Forest Fire Weather Index → 1D-CNN → GRU → Transformer → Improved Model (FWI‑MSNet) → Data Preprocessing → Multi‑scale Input Construction → Multi‑scale Feature Extraction Module → GRU‑Transformer Collaborative Mechanism. Second, we added more supporting references throughout the section: for the FWI equations (Refs. [23–29] in Section 2.1), 1D-CNN (Refs. [30–35] in Section 2.2), GRU (Refs. [36–41] in Section 2.3), Transformer (Refs. [42–45] in Section 2.4), and the collaborative mechanism (Refs. [46–49] in Section 2.5.4). All equations now have appropriate citations.

Third, we have clearly specified the packages and libraries used in each subsection. Section 2.5 states Python 3.9, PyTorch 1.10, NumPy, Pandas, and scikit‑learn; Section 2.5.1 mentions sklearn.preprocessing.StandardScaler; Section 2.5.2 notes Pandas and scikit‑learn’s StandardScaler; Section 2.5.3 specifies torch.nn for Conv1d, BatchNorm1d, ReLU, and Dropout; and Section 2.5.4 provides detailed hyperparameter tables (e.g., Table 5) reflecting the PyTorch implementations of GRU and Transformer. In addition, all key model parameters and architectural configurations (e.g., kernel sizes, hidden dimensions, number of attention heads, etc.) have been supplemented in Tables 4 and 5 within the corresponding subsections, further enhancing reproducibility. All changes are marked in red.

We are grateful for the reviewer’s insightful suggestions, which have significantly improved the comprehensiveness and depth of our Introduction.

**6. Ensure that each equation is supported with relevant references.**

***Author’s Reply:***

Thank you for this important requirement. In the revised manuscript, we have added a reference at the beginning of each formula block. Specifically: reference [29] supports Equations (1)–(7) in Section 2.1; [35] supports Equation (8) in Section 2.2; [41] supports Equations (9)–(12) in Section 2.3; [45] supports Equations (13)–(18) in Section 2.4; and [49] supports Equation (19), [50] supports Equation (20) in Section 2.5.3.

For Equations (21)–(25), which include Z‑score normalization and the evaluation metrics RMSE, MAE, R², and MAPE, these are standard formulas widely available in the literature. To maintain conciseness, we have not cited specific references for them in the manuscript.

We are grateful for the reviewer’s insightful suggestions, which have significantly improved the comprehensiveness and depth of our Introduction.

**7.** **For Table 6, please provide all the evaluation metrics separately for the training and testing datasets.**

***Author’s Reply:***

Thank you for this valuable suggestion. In the revised manuscript, we have provided the evaluation metrics separately for the training and testing datasets. Specifically, Table 8 (originally Table 6, but renumbered due to newly added tables earlier in the manuscript) presents all metrics for the training set, and Table 9 presents all metrics for the test set. Both tables are located in Section 4.3 (pages 31–32).

We are grateful for the reviewer’s insightful suggestions, which have significantly improved the comprehensiveness and depth of our Introduction.

**8.** **Add a Discussion section and mention the limitations of the current study.**

***Author’s Reply:***

Thank you for this important suggestion. In the revised manuscript, we have added a new Discussion section (now Section 5, starting on page 36), which explicitly discusses the limitations of the current study and outlines future research directions. Specifically, we point out three main limitations: (1) the model is trained only on data from a single ecological station in China, lacking spatial information such as vegetation indices and topographic features; (2) the current architecture does not model spatial fire propagation and has high computational complexity; (3) the Transformer’s self‑attention mechanism is not fully interpretable, and the model does not output intermediate physical states. Corresponding future work directions are also provided.

**9.** **The Conclusion needs improvement. Please clearly list the key findings of the study in the conclusion.**

***Author’s Reply:***

Thank you for this constructive suggestion. In the revised manuscript, we have thoroughly rewritten the Conclusion section (now Section 6, starting on page 37) to clearly list the key findings of the study. Specifically, we summarize five main findings: (1) superior prediction performance with detailed quantitative metrics (RMSE, MAE, R², MAPE); (2) effectiveness of physical alignment via FWI-based feature grouping; (3) multi-scale feature extraction using parallel 1D‑CNN with kernel sizes 7, 30, and 90; (4) temporal‑global fusion achieved by the GRU‑Transformer collaborative mechanism; and (5) case study validation on the 2020 Australian wildfires demonstrating generalizability.

We are grateful for the reviewer’s insightful suggestions, which have significantly improved the comprehensiveness and depth of our Introduction.

**10.** **Each table and figure in the Results section requires more complete explanation and discussion in the text.**

***Author’s Reply:***

Thank you for this important suggestion. In the revised manuscript, we have carefully reviewed every table and figure and added more complete explanations and discussions in the corresponding text. Specifically, we have expanded the description and analysis for Table 1 (page 6, Section 1), Table 2 (page 12, Section 2.5.2), Table 3 (page 13, Section 2.5.2), Table 4 (page 14, Section 2.5.3), Table 5 (page 19, Section 2.5.4), Table 6 (page 20, Section 3.1), Table 7 (page 24, Section 3.3), Table 8 (page 31, Section 4.3), Table 9 (page 32, Section 4.3), Table 10 (page 35, Section 4.4), as well as for Figure 1 (page 2, Section 1), Figure 2 (page 10, Section 2.5), Figure 3 (page 16, Section 2.5.3), Figure 4 (page 17, Section 2.5.4), Figure 5 (page 22, Section 3.3), Figure 6 (page 27, Section 4.2), Figure 7 (page 29, Section 4.3), Figure 8 (page 30, Section 4.3), Figure 9 (page 33, Section 4.3), and Figure 10 (page 34, Section 4.4). Each table and figure is now accompanied by a thorough discussion that highlights key observations, implications, and connections to the research objectives.

We are grateful for the reviewer’s insightful suggestions, which have significantly improved the comprehensiveness and depth of our Introduction.

**Reviewer #1:Reviewer Report**

**Recommendation**

**The topic is important and the pipeline is reasonable, but several key claims and evaluation details limit confidence. I recommend major revision to strengthen (a) the definition of the prediction task and inputs construction, (b) training leakage control, and (c) “physics-informed” claims in the title.**

***Author’s Reply:***

We thank the reviewer for summarizing the key concerns. We have carefully addressed all three points—(a) prediction task definition and input construction, (b) training leakage control, and (c) the “physics‑informed” claim—in the revised manuscript. Detailed point‑by‑point responses are provided below.

**1. “Physics-informed” is currently not demonstrated in the standard sense: The manuscript positions FWI as “the physical core” and describes the framework “physics-informed”. But the model appears to be a data-driven architecture guided by feature grouping rather than physics-informed optimization. The model does not explicitly incorporate physical constraints or differentiable governing equations. Please either rename the approach or add a true physics-informed component.**

***Author’s Reply:***

We sincerely thank the reviewer for this critical and insightful comment. We fully agree that the original use of the term “physics-informed” was inappropriate, as our model does not embed differentiable physical constraints or governing equations in the sense of Physics-Informed Neural Networks (PINNs). To avoid any misunderstanding, we have thoroughly revised the manuscript as follows:

First, we have renamed the approach throughout the paper. The new title is:

“Physically aligned Forest Fire Risk Prediction: A Deep Learning Framework Coupling Fuel and Climate Multivariate Factors”

All instances of “physics-informed” have been replaced with more accurate terms such as “physically aligned”, “feature‑guided”, or “data‑driven with physical feature grouping”. The term “physics‑informed” no longer appears in the manuscript.

Second, we have clarified that the model is aligned with physical principles through its architecture and feature organization, rather than through explicit differentiable constraints. Specifically:

The input features are grouped according to the multi‑scale temporal structure of the FWI system (short‑term climate‑driven, medium‑term eco‑hydrological, long‑term soil‑driven).

The kernel sizes of the parallel 1D‑CNN channels (7, 30, 90 days) are explicitly mapped to the characteristic timescales of FFMC, DMC, and DC dynamics, respectively.

The GRU‑Transformer collaborative mechanism captures both sequential dependencies and global contextual correlations, which are physically interpretable as fuel‑moisture evolution and climate‑anomaly modulation.

We believe that “physically aligned” accurately reflects the design philosophy of the proposed framework without overclaiming a true PINN implementation. All changes have been marked in red in the revised manuscript (see Title, Abstract, Introduction, and Section 2). We are grateful to the reviewer for helping us improve the terminological precision of our paper.

We are grateful for the reviewer’s insightful suggestions, which have significantly improved the comprehensiveness and depth of our Introduction.

**2. High risk of leakage in evaluation: The paper reports a random stratified split into train/val/test (70/15/15). For time series data this can introduce temporal leakage. Also, the model uses multi-scale convolutions with long-term path (up to a quarter), which could cause leakage. Please ensure that no overlapping windows cross split boundaries.**

***Author’s Reply:***

We thank the reviewer for raising this critical issue. We apologize for the confusion caused by the earlier description of the data split (70/15/15 random stratified split), which was a writing error and did not reflect the actual implementation. In fact, we used a chronological split of 80% training / 20% testing (no validation set was used separately). This has been corrected in the revised manuscript. Specifically, we have added a detailed description in Section 3.2 (page 21) as follows:

(1) Chronological split (no leakage): The first 80% of time‑ordered samples are used for training and the last 20% for testing. No future information leaks into training.

(2) No cross‑boundary windows: Sliding windows generate samples sequentially; the train/test split by index ensures temporal separation with no overlapping information.

(3) Multi‑scale convolution: Operates only within each sample; no cross‑sample interaction, no leakage.

(4) Safe augmentation: All augmentation (sliding window, multi‑scale sampling, noise) is performed within the original time series, preserving order and not introducing future information.

We are grateful for the reviewer’s insightful suggestions, which have significantly improved the comprehensiveness and depth of our Introduction.

**3. Sample construction and dataset description are unclear: The reported dataset size is inconsistent across sections. Also, please define at least one sample precisely, including the timestamp resolution, window length used to build features.**

***Author’s Reply:***

We apologize for the confusion. In the revised manuscript (Sections 2.5.1 and 3.3), we now clearly define a sample as 180 consecutive daily feature vectors (21 dimensions) with a daily timestamp resolution, used to predict the next 30 days of FWI values. The sliding window length is 180 days with a stride of 1 day.

Regarding the dataset size inconsistency: the raw data were unified to 216 monthly records, linearly interpolated to 6,570 daily records, and then processed via a sliding window (6,390 base sequences). Multi‑scale sampling (window lengths 90–240 days) and Gaussian noise augmentation (σ = 0.01, 0.02) were applied, finally yielding 33,560 samples. Other numbers (e.g., 2,214 samples in the GitHub demo) correspond to the same procedure using monthly data for quick testing.

We are grateful for the reviewer’s insightful suggestions, which have significantly improved the comprehensiveness and depth of our Introduction.

**4. Australia 2020 case study & Figure 10: clarify prediction vs ground truth: As presented, Figure 10 reads more like a descriptive time-series plot than “prediction vs reference” evaluation. Is the “Hourly FWI” FWI-MSNet predictions or computed from ERA5?**

***Author’s Reply:***

We thank the reviewer for catching this issue. In the original manuscript, the predicted value curve was inadvertently missing, and the label “Hourly FWI” referred only to the ground‑truth values (computed from ERA5 data). We have now updated Figure 10 (located in Section 4.4, page 34) to include both the predicted FWI (from FWI‑MSNet) and the ground‑truth FWI, with a clear legend distinguishing the two curves. The caption has also been revised accordingly.

**5. Section 4.1, “The Huitong Forest Ecological Station provides 18 years of continuous observations (2005-2012)…”, which is 8 years. Please correct.**

***Author’s Reply:***

We apologize for this typographical error. The correct time range is 2005–2022 (18 years). This has been corrected in Section 3.1 (page 19). All changes are marked in red.

**6. There are multiple grammar issues throughout. Please perform a careful English checking and improve clarity.**

***Author’s Reply:***

We have carefully proofread the entire manuscript to improve grammar and clarity. All changes are marked in red. Thank you for pointing this out.

**Reviewer #2:Reviewer Report**

**Recommendation**

**The paper has a strong basis as it integrates physics-informed indices with modern deep learning architectures. It has ablation studies and some baseline comparisons, and also presents a multi-scale modelling aligned with ecological processes.**

***Author’s Reply:***

We sincerely appreciate the reviewer’s positive assessment. It is encouraging to hear that the integration of physically aligned indices with deep learning, the ablation studies, and the multi‑scale ecological modeling are well recognized. Thank you for your constructive feedback throughout the review process. The specific revisions are detailed below.

**1. Improvement greater than 100% is unusual for regression metrics, and so stating that there is an "average accuracy improvement of approximately 120.7%" may be overstated, as RMSE reduction from 7.47 to 1.69 is 77% improvement. I think it would be a good idea for the researchers to explain the method used to calculate relative improvement.**

***Author’s Reply:***

We thank the reviewer for pointing out this error. The previously reported 120.7% figure was a mistake: it incorrectly included R² (which ranges from negative to 1) together with the error‑based metrics RMSE, MAE, and MAPE, leading to an inflated and misleading percentage. In the revised manuscript, we have corrected this. R² is now discussed separately, and the average improvement is recalculated using only RMSE, MAE, and MAPE, yielding approximately 56.1% (consistent with the values reported in the Abstract, Section 4.3, and Conclusions). The relative improvement calculation method (percentage reduction in error metrics) is clearly explained in Section 4.3 (pages 31–32).

We are grateful for the reviewer’s insightful suggestions, which have significantly improved the comprehensiveness and depth of our Introduction.

**2. There is a reference to "variant models and traditional models". Researchers should explicitly list CNNs, LSTMs, XGBoost, ablation models, and any other specific models they refer to.**

***Author’s Reply:***

Thank you for this constructive suggestion. In the revised manuscript, we have explicitly listed all compared models in Section 4.3 (page 28). Specifically, the traditional models include XGBoost, LSTM, and CNN; the ablation variants (referred to as “variant models”) include FWI-MSNet without multi-scale convolution, without Transformer, without GRU, and without FWI features. All models are clearly named and described in the text and tables.

**3. Even though they provide a good context for wildfire impacts, machine learning approaches and the need for integrated fuel-climate modelling, they do not clearly articulate the research gap. They can add a paragraph to the introduction that explicitly states the limitations of current ML wildfire prediction, the lack of physics-informed deep learning models, and the absence of multi-scale temporal modelling.**

***Author’s Reply:***

We thank the reviewer for this valuable suggestion. In the revised manuscript, we have added a dedicated paragraph in the Introduction (now Section 1, pages 3–4) that explicitly articulates the research gaps. Specifically, we point out three major limitations of existing approaches: (1) most deep learning models treat fire prediction as a purely data‑driven task, overlooking the inherent multi‑scale temporal structure of physical processes; (2) the Fire Weather Index (FWI) system has not been used as a physically aligned organizational principle for feature engineering; and (3) existing architectures struggle to simultaneously capture multi‑scale temporal patterns and long‑term dependencies in the coupled fuel‑climate system. These gaps are clearly stated before presenting the contributions of our work. All changes are marked in red.

We are grateful for the reviewer’s insightful suggestions, which have significantly improved the comprehensiveness and depth of our Introduction.

**4. It will be a good idea to include the datasets used, performance metrics and the limitations of the citations used under the literature review so that the section can have a bit more depth.**

***Author’s Reply:***

We thank the reviewer for this constructive suggestion. In the revised manuscript, we have enriched the literature review within the Introduction (Section 1, pages 2–5) by explicitly mentioning, for each cited work, the dataset used, the key performance metrics reported, and the main limitations of that approach. This additional information is integrated narratively into the existing text, providing readers with a deeper understanding of the current research landscape without breaking the flow of the introduction. We believe this significantly improves the depth and completeness of the literature review. All changes are marked in red.

We are grateful for the reviewer’s insightful suggestions, which have significantly improved the comprehensiveness and depth of our Introduction.

**5. An additional transformer description will help the depth of the paper. Add attention equation, positional encoding and architecture details.**

***Author’s Reply:***

Thank you for this valuable suggestion. In the revised manuscript, we have significantly expanded the description of the Transformer in Section 2.4 (pages 8–9). Specifically, we have added the scaled dot‑product attention equation (Equation 13), the multi‑head attention formulation (Equations 14–15), sinusoidal positional encoding equations (Equations 16–17), and the feed‑forward network equation (Equation 18), along with corresponding explanations.

Furthermore, detailed architecture parameters—including the number of attention heads (nhead=8), number of Transformer layers (num_layers=2), model dimension (d_model=128), feed‑forward dimension (dim_feedforward=512), and dropout rate (0.1)—are now provided in Table 5 (Section 2.5.4, page 18). These additions improve the depth and reproducibility of the Transformer component. All changes are marked in red.

We are grateful for the reviewer’s insightful suggestions, which have significantly improved the comprehensiveness and depth of our Introduction.

**6. On the model architecture, some critical information appears missing, including the number of GRU layers, hidden units, transformer heads, embedding dimension, dropout rate, optimiser, learning rate and batch size. Without these, reproducibility will be impossible.**

***Author’s Reply:***

We apologize for the omission. In the revised manuscript, all missing architectural and training hyperparameters have been clearly provided. Specifically, Table 5 (Section 2.5.4, page 1) now includes the number of GRU layers (num_layers=1), GRU hidden units (hidden_dim=128), Transformer heads (nhead=8), Transformer model dimension (d_model=128, equivalent to embedding dimension), dropout rates (0.1 for GRU and Transformer, 0.1 for output layer), and batch size (batch_size=32). The optimizer (Adam with an initial learning rate of 0.001) and the learning rate scheduling (cosine annealing) are described in Section 4.3 (page 28). All changes are marked in red, ensuring full reproducibility.

We are grateful for the reviewer’s insightful suggestions, which have significantly improved the comprehensiveness and depth of our Introduction.

R6. The key information of the model architecture has been provided.

**7. There is an inconsistent data time range "18 years of data (2005-2012)". This will affect the whole interpretation of data and should be rectified.**

***Author’s Reply:***

We apologize for this typographical error. The correct time range is 2005–2022 (18 years). This has been corrected in Section 3.1 (page 19). All changes are marked in red.

**8. The datasize is also unclear. The table shows soil observations of 4458, climate 216, moisture 168,962 and then a final dataset of 33,560 samples. Either they are explained, or the differences are made clear.**

***Author’s Reply:***

We apologize for the confusion. In the revised manuscript (Section 3.3, page 21), we have clearly defined a sample as 180 consecutive daily feature vectors (21 dimensions) used to predict the next day’s FWI (the prediction horizon is 30 days as stated in Section 4.3). The numbers 4,458 (soil), 216 (climate), and 168,962 (moisture) are the raw observation counts from the three original datasets before integration. After merging, unifying to monthly records (216), linear interpolation to daily records (6,570), and sliding window (L=180, stride=1), we obtain 6,390 base sequence samples. Through multi‑scale sampling (window lengths 90–240 days padded to 180) and Gaussian noise augmentation (σ = 0.01, 0.02), the final dataset contains 33,560 samples.

We are grateful for the reviewer’s insightful suggestions, which have significantly improved the comprehensiveness and depth of our Introduction.

**9. The paper claims "21 features selected based on correlation" but they do not specify the threshold, method or dimensionality reduction. They should also apply KNN imputation only on the training data and not the entire set so that data leakage doesn't occur.**

***Author’s Reply:***

We thank the reviewer for these important technical points. In the revised manuscript (Section 3.2, page 21), we have clarified that the 21 features were selected based on Pearson correlation with the target FWI using a threshold of |r| ≥ 0.6. No dimensionality reduction (e.g., PCA) was applied, as preserving the original physical meaning of each feature was essential for interpretability.

Regarding data leakage, we have corrected the imputation procedure: the dataset is first split chronologically into training (80%) and test (20%) sets; then the K‑nearest neighbor algorithm (k=5, Euclidean distance) is fitted only on the training set and applied to impute missing values in both training and test sets. This ensures no information from the test set is used during training. All changes are marked in red.

We are grateful for the reviewer’s insightful suggestions, which have significantly improved the comprehensiveness and depth of our Introduction.

**10. "with a time span of 30 days" makes it unclear whether the prediction horizon is 30 days or the training window is 30 days. Please clarify. Though the models used are listed, there is no information on hyperparameters, architecture and tuning procedure, and this may impact the fairness of the comparison.**

***Author’s Reply:***

We apologize for the ambiguity. In the revised manuscript, we have clarified that the prediction horizon is 30 days (i.e., the model outputs FWI values for the next 30 days), while the input window length is 180 days. This is now explicitly stated in Section 4.3 (page 28) and Section 3.3 (page 33).

Regarding the missing hyperparameters and tuning procedure: all relevant information has been provided. Table 5 (Section 2.5.4, page 19) lists the architecture details, including the number of GRU layers (num_layers=1), hidden units (hidden_dim=128), Transformer heads (nhead=8), embedding dimension (d_model=128), dropout rates (0.1 for GRU/Transformer, 0.1 for output layer), and batch size (batch_size=32). Section 4.3 (page 28) describes the optimizer (Adam, initial learning rate 0.001), loss function (MSE), early stopping (patience 20 epochs), and maximum epochs (200). For XGBoost, hyperparameters were grid‑searched (number of trees = 100, max depth = 6, learning rate = 0.1). All models were trained under identical conditions to ensure fair comparison. All changes are marked in red.

We are grateful for the reviewer’s insightful suggestions, which have significantly improved the comprehensiveness and depth of our Introduction.

**11. Professional proofreading is recommended as some phrases give unintended meanings and make reading the paper a bit tedious.**

***Author’s Reply:***

We have carefully proofread the entire manuscript to improve grammar and clarity. All changes are marked in red. Thank you for pointing this out.
