# Supplementary material for: Physically aligned forest fire risk prediction: A deep learning framework coupling fuel and climate multivariate factors
Source: PLoS One. 2026 Sep 1;21(9):e0355829. doi: 10.1371/journal.pone.0355829 (PMC13533387; doi:10.1371/journal.pone.0355829)
Supplement: Supplementary file 3 [file pone.0355829.s004.docx]

**Response to Reviewers**

Dear Editor and Reviewers,

We sincerely thank the Academic Editor and all reviewers for their thoughtful and constructive comments on our manuscript. We have carefully addressed each concern raised and provide a point‑by‑point response below. All changes in the revised manuscript have been highlighted for easy identification.

In response to the Additional Editor Comments, we have made the following key improvements: (1) added a Data Availability Statement with DOIs for the three source datasets, the GitHub repository link, and the ERA5 data source; (2) supplemented definitions for all key model variables and hyperparameters; (3) added a new subsection (Section 4.5) to explicitly compare the scientific insights of our deep learning framework with alternative models; (4) added two peer‑reviewed references (Wotton, 2009; Kudláčková et al., 2024) to supplement the original technical report; (5) clarified the model‘s contribution and why it improves predictions in Section 4.5; and (6) improved all figures using the NAAS tool to meet PLOS ONE’s technical requirements.

We are grateful to the editor for pointing out the figure numbering issue in the case study figure caption of Section 4.4. In response, we have corrected the figure number in the caption from “Fig 1” to “Fig 11” to match the in‑text citation.

We have also responded to all reviewer comments, including those from Reviewer #2 (positive feedback), Reviewer #3 (eight specific points), and Reviewer #4 (no specific comments). All revisions have been made to the manuscript accordingly. We believe the revised version now meets the publication standards of PLOS ONE.

**Additional Editor Comments:**

**Omissions in the paper:**

**1. key datasets are not provided.**

***Author’s Reply:***

We thank the editor for this valuable suggestion. In the revised manuscript, we have added a dedicated Data Availability Statement (placed before the References) that clearly:

(1) lists the three source datasets with their DOI links from the China Ecological Science Data Center;

(2) explains that the full datasets are subject to third-party data usage agreements and are accessible through the data center;

(3) provides the link to our GitHub repository, which contains the complete source code and a demo dataset for readers to test the model;

(4) cites the ERA5 data source for the case study.

We believe these additions fully address the data availability concern. We sincerely appreciate the editor’s time and thoughtful guidance in helping us improve the transparency and reproducibility of our work.

**2. important variables used in the model are not defined or explained.**

***Author’s Reply:***

We thank the reviewer for this valuable comment. Upon careful review, we found that most of the key variables and hyperparameters used in our model were already defined and explained in the original manuscript. However, we also identified that the data preprocessing parameters in Table 2 lacked explicit justification. To address this, we have added a dedicated paragraph after Table 2 to explain these parameters, including the choice of sequence length (6 months), stride (1), noise levels (0.01 and 0.02), and the 80/20 chronological split.

Specifically:

FWI system variables (FFMC, DMC, DC, ISI, BUI, FWI) and their physical meanings were already provided in Section 2.1.

Multi-scale CNN parameters (kernel sizes 7, 30, 90 and out_channels 32) were already explained in Section 2.5.3.

GRU-Transformer hyperparameters (hidden_dim, nhead, num_layers, etc.) were already explained in Section 2.5.4.

Data preprocessing parameters (base_seq_length, stride, noise_levels, train_ratio, test_ratio) — we have now added explicit explanations for these in a new paragraph after Table 2.

We believe these additions and clarifications fully address the reviewer's concern. We sincerely appreciate the reviewer’s valuable suggestion, which has helped us improve the clarity of our manuscript.

**3. the insights provided by deep learning are not compared to other model approaches.**

***Author’s Reply:***

We thank the reviewer for this constructive suggestion. In the revised manuscript, we have added a new subsection in the Discussion (Section 4.5) that explicitly compares the scientific insights derived from our deep learning framework with those from alternative model approaches.

Specifically, based on the experimental results reported in Table 9, we highlight three key insights revealed by FWI-MSNet that traditional models (e.g., XGBoost, LSTM, CNN) cannot provide:

(1) The FWI system serves as a critical physical anchor for fire risk prediction. Removing FWI features degrades R² from 0.9251 to 0.5115 (a 44.7% decrease relative to the full model), demonstrating that the FWI system is not merely an additional input variable but a foundational physical prior. Traditional models that treat all inputs uniformly cannot reveal this distinction.

(2) The simultaneous modeling of daily, monthly, and seasonal scales is essential. Removing multi-scale convolution increases RMSE from 1.6892 to 2.4227 (a 43.4% increase, based on Table 9). This indicates that the fuel-climate coupling mechanism operates across multiple temporal scales simultaneously, and a single-scale model inevitably misses critical information at other scales.

(3) GRU and Transformer play distinct but complementary roles. Removing GRU increases RMSE to 2.4466, while removing Transformer increases it to 3.0047 (Table 9). This decomposition reveals that GRU primarily captures local sequential evolution of fuel moisture, whereas Transformer captures global cross-period correlations (e.g., remote modulation by climate anomalies).

We believe these additions, derived directly from the ablation experiments in Table 9, demonstrate that our physically aligned deep learning framework offers unique interpretable insights into the multi-scale fuel-climate coupling mechanism—a capability that conventional black-box models lack. We sincerely appreciate the reviewer's insightful suggestion, which has helped us better articulate the unique value of our deep learning framework and strengthened the overall discussion of our findings. We believe this addition fully addresses the reviewer's concern.

**4. key literature citations are unpublished reports.**

***Author’s Reply:***

We thank the reviewer for this careful observation. The reviewer is referring to Ref. [29] (Van Wagner & Pickett, 1985), which is a Technical Report from the Canadian Forestry Service. We cited this report because it is the original and definitive source that documents the complete mathematical formulation of the FWI system—the core physical foundation of our model. This report remains the standard reference for FWI equations in the fire research community.

However, we agree with the reviewer that supplementing this technical report with peer-reviewed literature strengthens the paper. In the revised manuscript, we have added two peer-reviewed references on the FWI system:

Wotton, B.M. (2009) — Interpreting and using outputs from the Canadian Forest Fire Danger Rating System in research applications. Environmental and Ecological Statistics, 16(2), 107–131. This reference provides a comprehensive, peer-reviewed overview of the FWI system and its outputs, serving as an authoritative supplement to the original technical report.

Kudláčková, L., et al. (2024) — Assessing fire danger classes and extreme thresholds of the Canadian Fire Weather Index across global environmental zones: a review. Environmental Research Letters, 20(1), 013001. This recent review synthesizes findings from 750 studies across 18 global environmental zones, demonstrating the widespread applicability and effectiveness of the FWI system.

Both references have been cited in Section 2.1 alongside the original Technical Report [29], ensuring that readers have access to both the foundational mathematical formulation and supporting peer-reviewed evidence. We sincerely appreciate the reviewer's valuable suggestion, which has helped us strengthen the literature foundation of our manuscript.

**5. The third reviewer raises issues, which were also raised by the first two reviewers. In their revision, the authors did not address the key questions of all reviewers, which are, what does this model (deep learning) with a fire weather index contribute, and why does it improve fire risk predictions?**

***Author’s Reply:***

We sincerely thank the editor for this critical comment. To directly address the key questions raised by all reviewers—what does this model contribute, and why does it improve fire risk predictions?—we have added a new subsection in the Discussion (Section 4.5) titled “Insights from Deep Learning Compared to Alternative Models.”

In this subsection, we explicitly articulate that our contribution lies not in proposing a new deep learning architecture, but in establishing a physically aligned framework that uses the FWI system as an organizational principle for feature engineering. Specifically, we demonstrate three key insights based on the quantitative results reported in Table 9:

The FWI system provides a critical physical anchor. Removing FWI features degrades R² from 0.9251 to 0.5115, a 44.7% decrease.

Multi-scale modeling is essential. Removing multi-scale convolution increases RMSE from 1.6892 to 2.4227, a 43.4% increase.

GRU and Transformer play distinct but complementary roles. Removing GRU increases RMSE to 2.4466, while removing Transformer increases it to 3.0047.

These findings demonstrate that FWI-MSNet improves predictions by aligning the model architecture with the multi-scale physical structure of the fuel-climate coupling mechanism—an approach that conventional black-box models (XGBoost, LSTM, CNN) cannot provide. We have added this new subsection to the revised manuscript (Section 4.5). We believe this addition fully addresses the reviewers' concern. We sincerely appreciate the editor's and reviewers' valuable guidance, which has helped us significantly improve the clarity of our contribution.

**6. The figures are of poor quality.**

***Author’s Reply:***

We thank the editor for raising this concern. In the revised manuscript, we have thoroughly improved all figures to meet PLOS ONE's technical requirements.

Specifically, all eleven figures were uploaded to and checked using the NAAS digital diagnostic tool (https://ngplosjournals.pagemajik.ai/artanalysis), the official pre-submission image quality checker recommended by PLOS ONE. The tool confirmed that ten of the eleven figures passed the inspection and were adjusted to meet the specifications, including resolution adjustment to 300 DPI where needed, dimensional adjustment to within the required width limit (≤ 7.5 inches), and conversion to valid TIFF format. For Figure 7, the tool indicated that it could not automatically adapt the image; we manually adjusted its width to 7.5 inches with 300 DPI resolution and re-exported it as a TIFF file. The corrected version now fully complies with PLOS ONE's specifications.

We believe all figures now meet the publication standards of PLOS ONE. We sincerely appreciate the editor's attention to this important presentation detail.

**Failure to meet PLOS One criteria for papers that describe new methods or software for applications:**

**1. utility: PLOS One requirement: “The tool must be of use to the community and must present a proven advantage over existing alternatives, where applicable.“**

**The model presented in the paper performs well because of the incorporation of a fire weather index. However, the manuscript does not explain how the fire weather index is calculated, why deep learning using the fire weather index improves model performance, or how their use of the fire weather index in deep learning is different from other models. The fire weather index citations are two unpublished reports from Canada. It is not explained how field data were used to create a fire weather index.**

***Author’s Reply:***

We thank the editor for this comment. We have carefully addressed each of the three concerns raised.

First, regarding the calculation of the FWI: The FWI system and its complete mathematical formulations are already provided in Section 2.1 (Equations 1–7), where all six components (FFMC, DMC, DC, ISI, BUI, and FWI) are defined with their governing equations. Additionally, Section 3.2 describes how the FWI components were calculated from the field observations (temperature, relative humidity, wind speed, and precipitation) on a daily basis. Regarding the citation: we have retained Ref. [29] (Van Wagner & Pickett, 1985) as the original source of the FWI equations, and have supplemented it with two peer-reviewed references [56, 57] in the revised manuscript.

Second, regarding why deep learning with FWI improves performance: We have added a new subsection in the Discussion (Section 4.5) that explicitly explains the mechanisms. Based on the ablation results in Table 9, we demonstrate that the FWI system serves as a physical anchor—removing FWI features degrades R² from 0.9251 to 0.5115 (a 44.7% decrease). This confirms that the FWI system is not merely an additional input but a foundational physical prior that constrains and guides the learning process.

Third, regarding how our use of FWI differs from other models: We have clarified this in both the Introduction and Section 4.5. Unlike conventional approaches that treat FWI components merely as additional input variables, our framework uses the FWI system as an organizational principle for feature engineering. Specifically, we explicitly map the FWI‘s inherent multi‑scale temporal structure (FFMC—daily, DMC—monthly, DC—seasonal) to the parallel 1D‑CNN kernel sizes (7, 30, and 90 days). This ensures that the learning process operates at the same temporal scales as the physical processes governing fire risk formation—a design that conventional black‑box models lack.

We believe these clarifications fully address the editor’s concerns. We sincerely appreciate the editor’s valuable guidance, which has helped us better articulate the utility of our framework.

**2. validation: PLOSone requires that ““Submissions presenting methods, software, databases, or tools must demonstrate that the new tool achieves its intended purpose. If similar options already exist, the submitted manuscript must demonstrate that the new tool is an improvement over existing options in some way. This requirement may be met by including a proof-of-principle experiment or analysis;”**

**A validation is performed for a case study of fire in Australia in 2020, but it is not explained. No citation is provided for the data used for the case study in Australia, either in the literature cited or the data availability statement. The validation results are shown in Figure 10 but only as a fire weather index, and the calculation of the fire weather index is not defined or explained in the paper.**

***Author’s Reply:***

We thank the editor for this comment. In the revised manuscript, we have made the following clarifications to address each of the concerns raised.

First, regarding the citation for the Australian case study data: The ERA5 reanalysis data used for the case study was already cited as Ref. [55] in the original manuscript. In the revised version, we have also explicitly listed this dataset in the Data Availability Statement to ensure readers can clearly identify the data source.

Second, regarding the calculation of the FWI for the case study: The FWI calculation follows the same mathematical formulations already defined in Section 2.1 (Equations 1–7). To make this explicit, we have added the following sentence at the beginning of Section 4.4:

“The FWI values for the case study are calculated using the same mathematical formulations described in Section 2.1, driven by ERA5 meteorological inputs (temperature, relative humidity, wind speed, and precipitation).”

Third, regarding the case study description: The case study setup, including the six representative regions across Australia (East Gippsland, Canberra, Blue Mountains, Melbourne, South Coast, and Sydney Area), is already described in Section 4.4. The validation results are quantitatively reported in Table 10, with R² values ranging from 0.57 to 0.77 and MAPE approximately 25% across all regions. This demonstrates cross-regional generalization performance on unseen data from a different continent, confirming the model’s practical utility beyond the training region. The model performs best in forest-dominated areas (R² > 0.76) and shows consistent tracking of extreme fire risk transitions, as illustrated in Figure 10.

We believe these additions fully address the editor’s concerns. We sincerely appreciate the editor’s valuable guidance, which has helped us improve the transparency of our validation.

**3. availability: PLOS One requirement: “If the manuscript’s primary purpose is the description of new software or a new software package, this software must be open source, deposited in an appropriate archive, and conform to the Open Source Definition.“**

**The data availability statement does not provide any links to data. The model software is not available. There are no links provided to key datasets, such as the fire weather index, which was central to their model. The only datasets provided are for meteorological, water, and soil data from the National Research Station of Huitong Forest Ecosystems and some ERA5 data.**

***Author’s Reply:***

We thank the editor for this comment. We have carefully addressed the availability concern in the revised manuscript.

First, regarding the model software: The complete source code of FWI-MSNet is publicly available at the following GitHub repository: https://github.com/CBJYB/FWIMSNet-Forest-Fire-Risk-Prediction-Framework. This repository contains all scripts necessary to reproduce the model training, evaluation, and prediction.

Second, regarding the data: For readers to test the code and reproduce the results, we have provided a demo dataset (216 monthly records) in the same GitHub repository, which follows the same preprocessing pipeline as the full dataset. For researchers who wish to access the complete dataset, we have added a dedicated Data Availability Statement in the revised manuscript that provides the DOIs for the three source datasets from the China Ecological Science Data Center:

1.Climate data: https://doi.org/10.12199/nesdc.ecodb.2021YFF0703900.htf.2025.1

2.Water environment data: https://doi.org/10.12199/nesdc.ecodb.2021YFF0703900.htf.2025.2

3.Soil data: https://doi.org/10.12199/nesdc.ecodb.2021YFF0703900.htf.2025.3

Due to third-party data usage agreements with the China Ecological Science Data Center, the full datasets are not publicly redistributed by the authors. However, they can be accessed by qualified researchers upon reasonable request to the data center via the DOIs provided above, or by contacting the corresponding author for guidance on data access procedures.

Third, regarding the ERA5 data: The ERA5 reanalysis data used for the Australian case study are openly available from the Copernicus Climate Change Service: https://doi.org/10.24381/cds.adbb2d47.

We believe these additions fully address the editor‘s availability concern. We sincerely appreciate the editor’s valuable guidance, which has helped us significantly improve the transparency and reproducibility of our work.

**Review Comments to the Author**

**Reviewer #2:**

**I can see extensive work has been done, and the document has been considerably improved. The Dataset has now been made available, and the abstract and methodology sections have been revised as advised. References have also been updated with more current literature.**

***Author’s Reply:***

We sincerely thank Reviewer #2 for the positive and encouraging feedback. We are delighted to hear that our revisions have been well received. We appreciate the reviewer’s valuable suggestions throughout the review process, which have significantly helped us improve the quality of our manuscript. We are grateful for the time and effort the reviewer has dedicated to evaluating our work.

**Reviewer #3:**

**1. The writing of the paper needs a lot of improvement in terms of grammar, spelling, and presentation.**

***Author’s Reply:***

We sincerely thank the reviewer for this valuable comment. We acknowledge that the language quality of our manuscript needed improvement. To address this, we have carefully revised the entire manuscript with a focus on grammar, spelling, and overall presentation.

Specifically, we have:

Conducted a thorough language review throughout the manuscript to correct grammatical errors and improve sentence structure

Refined unclear expressions and redundant descriptions to enhance readability and precision

Standardized terminology to ensure consistency across all sections

We believe these revisions have substantially improved the clarity and readability of the manuscript. We sincerely appreciate the reviewer's attention to this important aspect, which has helped us enhance the overall quality of our presentation.

**2. What is the novelty of this paper?**

***Author’s Reply:***

We thank the reviewer for this important question. In the revised manuscript, we have clarified the novelty of our work in two places: at the end of the Introduction and in the newly added Section 4.5 (Discussion) .

The novelty of this framework lies in three aspects:

(1) Physically aligned feature organization. Unlike conventional approaches that treat FWI components merely as additional input variables, our framework organizes the 21 input features according to the FWI system‘s inherent multi-scale temporal structure: short-term (FFMC, daily), medium-term (DMC, monthly), and long-term (DC, seasonal). This ensures that the learning process operates at the same temporal scales as the physical processes governing fire risk formation.

(2) Explicit physical mapping in architecture design. We design parallel 1D‑CNN branches with kernel sizes (7, 30, and 90 days) that directly correspond to the intrinsic time scales of the FWI components. This explicit mapping embeds domain knowledge into the network structure, grounding the learning process in physical principles rather than relying solely on black‑box optimization.

(3) GRU-Transformer collaborative mechanism for temporal-global fusion. We integrate GRU and Transformer to capture both local sequential evolution (GRU) and global cross-period correlations (Transformer), enabling a comprehensive representation of the coupled fuel-climate dynamics.

The effectiveness of this physical alignment is quantitatively validated by our ablation experiments (Table 9), which show that removing FWI features degrades R² from 0.9251 to 0.5115 (a 44.7% decrease). We have clarified this novelty in the Introduction and further elaborated in Section 4.5. We believe this fully addresses the reviewer‘s concern. We sincerely appreciate the reviewer’s valuable question, which has helped us better articulate the unique contribution of our work.

**3. Add a flowchart to show what is done in this study.**

***Author’s Reply:***

We thank the reviewer for this helpful suggestion. In the revised manuscript, we have added a flowchart (new Fig 2) that clearly illustrates the overall workflow of this study. The figure is placed at the beginning of the Methodology section, positioned between the section heading and Section 2.1, to provide readers with an overview of the entire study before they encounter the detailed technical descriptions.

As shown in Fig 2, the workflow consists of four main stages: (1) Data Preparation, including data cleaning, FWI calculation, standardization, and augmentation; (2) Multi-scale Feature Construction, where 21 features are organized into three groups (short-term, medium-term, and long-term) based on the FWI system‘s temporal structure; (3) Model Construction, consisting of parallel multi-scale 1D-CNN, feature concatenation, GRU-Transformer collaboration, and fully connected output layer; and (4) Model Evaluation & Validation, including ablation experiments, comparisons with traditional models, and case study validation.

The accompanying caption in the manuscript reads:

Fig 2. Overall workflow of this study. The workflow consists of four main stages: (1) Data Preparation, including data cleaning, FWI calculation, standardization, and augmentation; (2) Multi-scale Feature Construction, where 21 features are organized into three groups (short-term, medium-term, long-term) based on the FWI system‘s temporal structure; (3) Model Construction, consisting of parallel multi-scale 1D-CNN, feature concatenation, GRU-Transformer collaboration, and fully connected output layer; and (4) Model Evaluation & Validation, including ablation experiments, comparisons with traditional models, and case study validation. Detailed parameter settings are provided in Tables 2–5 and Section 4.1.

We believe this addition significantly improves the clarity and readability of the paper by providing readers with a clear roadmap of the study before they delve into the detailed methodological descriptions. We sincerely appreciate the reviewer’s valuable suggestion.

**4. Most Figures are blurred.**

***Author’s Reply:***

We thank the reviewer for pointing out this issue. In the revised manuscript, we have thoroughly improved all figures to meet PLOS ONE's technical requirements.

Specifically, all eleven figures were uploaded to and checked using the NAAS digital diagnostic tool (https://ngplosjournals.pagemajik.ai/artanalysis), the official pre-submission image quality checker recommended by PLOS ONE. The tool confirmed that ten of the eleven figures passed the inspection and were adjusted to meet the specifications, including resolution adjustment to 300 DPI where needed, dimensional adjustment to within the required width limit (≤ 7.5 inches), and conversion to valid TIFF format. For Figure 7, the tool indicated that it could not automatically adapt the image; we manually adjusted its width to 7.5 inches with 300 DPI resolution and re-exported it as a TIFF file. The corrected version now fully complies with PLOS ONE's specifications.

We believe all figures now meet the publication standards of PLOS ONE. We sincerely appreciate the reviewer's attention to this important presentation detail.

**5. The mathematics used throughout the article is still not very strict. Please try to update and illustrate some elements in the mathematical model that are not defined very strictly.**

***Author’s Reply:***

We thank the reviewer for this valuable comment. In the revised manuscript, we have carefully reviewed all mathematical expressions and ensured that every symbol and variable is rigorously defined at its first appearance.

Specifically, we have supplemented the definitions of all variables in the FWI system (Section 2.1), the 1D-CNN convolution operation (Section 2.2), the GRU update and reset gate mechanisms (Section 2.3), and the Transformer attention and positional encoding formulations (Section 2.4). In addition, we have added a formal derivation of the complete information flow in the GRU-Transformer collaborative mechanism (Section 2.5.4), describing the sequential transformation from multi-scale feature extraction (CNN) and temporal modeling (GRU) to global dependency learning (Transformer) and final prediction (fully connected layer).

We believe these revisions have significantly improved the mathematical rigor of the paper. We sincerely appreciate the reviewer's valuable suggestion.

**6. The result part is week, results and discussion should be better explained.**

***Author’s Reply:***

We thank the reviewer for this valuable suggestion. In the revised manuscript, we have strengthened the results and discussion section in the following ways:

First, we have added a new subsection Section 4.5 (Insights from Deep Learning Compared to Alternative Models) , which explicitly interprets the experimental results beyond numerical comparisons. Based on the ablation results in Table 9, we discuss three key findings:

(1) the FWI system serves as a physical anchor—removing it degrades R² from 0.9251 to 0.5115 (a 44.7% decrease);

(2) multi-scale modeling is essential—removing multi-scale convolution increases RMSE by 43.4%;

(3) GRU and Transformer play distinct but complementary roles—GRU captures local sequential evolution while Transformer captures global cross-period correlations.

Second, we have expanded the explanations in Section 4.3 (comparative experiments) and Section 4.4 (case study) to provide clearer interpretations of why FWI-MSNet outperforms the baseline models and how the physically aligned design contributes to the improved performance. For the case study, we have also clarified that the FWI values are calculated using the same formulations described in Section 2.1, driven by ERA5 meteorological inputs.

Third, in Section 5 (Discussion) , we have explicitly connected the quantitative results to the broader implications for fire risk prediction, highlighting the practical value of the model's interpretability and cross-regional generalization capability.

We believe these revisions have substantially improved the depth and clarity of the results and discussion. We sincerely appreciate the reviewer's valuable suggestion.

**7. Revise the conclusions and make it concise, (Better to write the main outcomes)**

***Author’s Reply:***

We thank the reviewer for this suggestion. In the revised manuscript, we have condensed the conclusions from five points to three, focusing only on the main outcomes: prediction performance, effectiveness of physical alignment, and case study validation. The detailed descriptions of the multi-scale feature extraction and GRU-Transformer fusion mechanisms have been removed to improve conciseness. We believe the revised conclusions now meet the reviewer‘s expectation. We sincerely appreciate the reviewer’s valuable suggestion.

**8. Include these Key Suggested References:**

**https://doi.org/10.1038/s41598-025-14908-x**

**DOI 10.21608/SVUSRC.2024.308832.1232**

**https://doi.org/10.58491/2735-4202.3263**

**https://doi.org/10.1038/s41598-025-16828-2**

**https://doi.org/10.1038/s41598-025-87208-z**

***Author’s Reply:***

We thank the reviewer for suggesting these references. We have carefully reviewed each of the five papers. Among them, we found that one paper — the comparative study of deep learning architectures for time series prediction (Scientific Reports, 2025) — is methodologically relevant to our work, as it evaluates the performance of CNN, LSTM, GRU, and Transformer models for time series forecasting. We have added this reference in the Introduction (now cited as Ref. [58]) where we discuss deep learning approaches for time series prediction.

The remaining four papers focus on topics unrelated to our study: wireless optical communication systems, photovoltaic panel surface classification, damage detection in photovoltaic panels, and grid-connected PV/wind systems with fuzzy control. These are outside the scope of our forest fire risk prediction research. Therefore, we have not included them in the revised manuscript.

We sincerely appreciate the reviewer's effort to provide these suggestions.
