# Supplementary material for: Physically aligned forest fire risk prediction: A deep learning framework coupling fuel and climate multivariate factors
Source: PLoS One. 2026 Sep 1;21(9):e0355829. doi: 10.1371/journal.pone.0355829 (PMC13533387; doi:10.1371/journal.pone.0355829)
Supplement: Peer review history

# Peer review: Decision Letter 0

**DOI:** 10.1371/journal.pone.0355829.r001

**Authors:** Babak Mohammadi

12 Mar 2026

Dear Dr. Zeng,

1. The manuscript proposes a deep learning framework; however, the source code and access to this framework are not provided. Please provide the source code of the developed framework for journal readers. You may upload it to a repository or upload center and provide its link in the methodology section.

2. Restructure the methodology based on the provided source code to present a step-by-step guideline for journal readers showing how to use this framework.

3. Sections 2 and 3 can be merged into a single section titled Methodology.

4. The Introduction requires substantial revision in terms of both quality and quantity. Please include a more comprehensive literature review, clearly define the research gaps, and then highlight the novelty of the current study.

5. The methodology section (currently Sections 2 and 3) needs better organization. Please restructure it based on the newly added source code. These sections also require more supporting references. In addition, each subsection should specify which packages or libraries were used.

6. Ensure that each equation is supported with relevant references.

7. For Table 6, please provide all the evaluation metrics separately for the training and testing datasets.

8. Add a Discussion section and mention the limitations of the current study.

9. The Conclusion needs improvement. Please clearly list the key findings of the study in the conclusion.

10. Each table and figure in the Results section requires more complete explanation and discussion in the text.

Please submit your revised manuscript by Apr 26 2026 11:59PM. If you will need more time than this to complete your revisions, please reply to this message or contact the journal office at plosone@plos.org. When you're ready to submit your revision, log on to https://www.editorialmanager.com/pone/ and select the 'Submissions Needing Revision' folder to locate your manuscript file.

- A letter that responds to each point raised by the academic editor and reviewer(s). You should upload this letter as a separate file labeled 'Response to Reviewers'.
- A marked-up copy of your manuscript that highlights changes made to the original version. You should upload this as a separate file labeled 'Revised Manuscript with Track Changes'.
- An unmarked version of your revised paper without tracked changes. You should upload this as a separate file labeled 'Manuscript'.

If you would like to make changes to your financial disclosure, please include your updated statement in your cover letter. Guidelines for resubmitting your figure files are available below the reviewer comments at the end of this letter.

If applicable, we recommend that you deposit your laboratory protocols in protocols.io to enhance the reproducibility of your results. Protocols.io assigns your protocol its own identifier (DOI) so that it can be cited independently in the future. For instructions see: https://journals.plos.org/plosone/s/submission-guidelines#loc-laboratory-protocols. Additionally, PLOS ONE offers an option for publishing peer-reviewed Lab Protocol articles, which describe protocols hosted on protocols.io. Read more information on sharing protocols at https://plos.org/protocols?utm_medium=editorial-email&utm_source=authorletters&utm_campaign=protocols.

We look forward to receiving your revised manuscript.

Kind regards,

Babak Mohammadi

Academic Editor

PLOS One

Journal Requirements:

When submitting your revision, we need you to address these additional requirements.

1. Please ensure that your manuscript meets PLOS ONE's style requirements, including those for file naming. The PLOS ONE style templates can be found at

https://journals.plos.org/plosone/s/file?id=wjVg/PLOSOne_formatting_sample_main_body.pdf and https://journals.plos.org/plosone/s/file?id=ba62/PLOSOne_formatting_sample_title_authors_affiliations.pdf

2. Please note that PLOS One has specific guidelines on code sharing for submissions in which author-generated code underpins the findings in the manuscript. In these cases, we expect all author-generated code to be made available without restrictions upon publication of the work. Please review our guidelines at https://journals.plos.org/plosone/s/materials-and-software-sharing#loc-sharing-code and ensure that your code is shared in a way that follows best practice and facilitates reproducibility and reuse.

3. Thank you for stating in your Funding Statement:

[Funding:This work was supported by a Research Project Grant from Yibin University (No. 412-2024XJPY06).].

Please provide an amended statement that declares *all* the funding or sources of support (whether external or internal to your organization) received during this study, as detailed online in our guide for authors at http://journals.plos.org/plosone/s/submit-now.  Please also include the statement “There was no additional external funding received for this study.” in your updated Funding Statement.

Please include your amended Funding Statement within your cover letter. We will change the online submission form on your behalf.

4. Thank you for stating the following in your manuscript:

[This work was supported by a Research Project Grant from Yibin University (No. 412-2024XJPY06).]

We note that you have provided funding information that is currently declared in your Funding Statement. However, funding information should not appear in the Acknowledgments section or other areas of your manuscript. We will only publish funding information present in the Funding Statement section of the online submission form.

Please remove any funding-related text from the manuscript and let us know how you would like to update your Funding Statement. Currently, your Funding Statement reads as follows:

[Funding:This work was supported by a Research Project Grant from Yibin University (No. 412-2024XJPY06).]

Please include your amended statements within your cover letter; we will change the online submission form on your behalf.

5. PLOS requires an ORCID iD for the corresponding author in Editorial Manager on papers submitted after December 6th, 2016. Please ensure that you have an ORCID iD and that it is validated in Editorial Manager. To do this, go to ‘Update my Information’ (in the upper left-hand corner of the main menu), and click on the Fetch/Validate link next to the ORCID field. This will take you to the ORCID site and allow you to create a new iD or authenticate a pre-existing iD in Editorial Manager.

6. Please include a separate caption for each figure in your manuscript.

If the reviewer comments include a recommendation to cite specific previously published works, please review and evaluate these publications to determine whether they are relevant and should be cited. There is no requirement to cite these works unless the editor has indicated otherwise.

Additional Editor Comments:

1. The manuscript proposes a deep learning framework; however, the source code and access to this framework are not provided. Please provide the source code of the developed framework for journal readers. You may upload it to a repository or upload center and provide its link in the methodology section.

2. Restructure the methodology based on the provided source code to present a step-by-step guideline for journal readers showing how to use this framework.

3. Sections 2 and 3 can be merged into a single section titled Methodology.

4. The Introduction requires substantial revision in terms of both quality and quantity. Please include a more comprehensive literature review, clearly define the research gaps, and then highlight the novelty of the current study.

5. The methodology section (currently Sections 2 and 3) needs better organization. Please restructure it based on the newly added source code. These sections also require more supporting references. In addition, each subsection should specify which packages or libraries were used.

6. Ensure that each equation is supported with relevant references.

7. For Table 6, please provide all the evaluation metrics separately for the training and testing datasets.

8. Add a Discussion section and mention the limitations of the current study.

9. The Conclusion needs improvement. Please clearly list the key findings of the study in the conclusion.

10. Each table and figure in the Results section requires more complete explanation and discussion in the text.

[Note: HTML markup is below. Please do not edit.]

Reviewers' comments:

Reviewer's Responses to Questions

**Comments to the Author**

1. Is the manuscript technically sound, and do the data support the conclusions?

Reviewer #1: Partly

Reviewer #2: Partly

**********

2. Has the statistical analysis been performed appropriately and rigorously?

Reviewer #1: I Don't Know

Reviewer #2: No

**********

3. Have the authors made all data underlying the findings in their manuscript fully available?

The PLOS Data policy

Reviewer #1: Yes

Reviewer #2: Yes

**********

4. Is the manuscript presented in an intelligible fashion and written in standard English?

Reviewer #1: No

Reviewer #2: No

**********

Reviewer #1: Reviewer Report

Manuscript Title:

“Physics-Informed Forest Fire Risk Prediction: A Deep Learning Framework Coupling Fuel and Climate Multivariate Factors”

Recommendation

The topic is important and the pipeline is reasonable, but several key claims and evaluation details limit confidence. I recommend major revision to strengthen (a) the definition of the prediction task and inputs construction, (b) training leakage control, and (c) “physics-informed” claims in the title.

Major Comments

1. “Physics-informed” is currently not demonstrated in the standard sense: The manuscript positions FWI as “the physical core” and describes the framework “physics-informed”. But the model appears to be a data-driven architecture guided by feature grouping rather than physics-informed optimization. The model does not explicitly incorporate physical constraints or differentiable governing equations. Please either rename the approach or add a true physics-informed component.

2. High risk of leakage in evaluation: The paper reports a random stratified split into train/val/test (70/15/15). For time series data this can introduce temporal leakage. Also, the model uses multi-scale convolutions with long-term path (up to a quarter), which could cause leakage. Please ensure that no overlapping windows cross split boundaries.

3. Sample construction and dataset description are unclear: The reported dataset size is inconsistent across sections. Also, please define at least one sample precisely, including the timestamp resolution, window length used to build features.

4. Australia 2020 case study & Figure 10: clarify prediction vs ground truth: As presented, Figure 10 reads more like a descriptive time-series plot than “prediction vs reference” evaluation. Is the “Hourly FWI” FWI-MSNet predictions or computed from ERA5?

Minor Comments

1) Section 4.1, “The Huitong Forest Ecological Station provides 18 years of continuous observations (2005-2012)…”, which is 8 years. Please correct.

2) There are multiple grammar issues throughout. Please perform a careful English checking and improve clarity.

Reviewer #2: The paper has a strong basis as it integrates physics-informed indices with modern deep learning architectures. It has ablation studies and some baseline comparisons, and also presents a multi-scale modelling aligned with ecological processes.

There are, however, some major concerns.

1. Improvement greater than 100% is unusual for regression metrics, and so stating that there is an "average accuracy improvement of approximately 120.7%" may be overstated, as RMSE reduction from 7.47 to 1.69 is 77% improvement. I think it would be a good idea for the researchers to explain the method used to calculate relative improvement.

2. There is a reference to "variant models and traditional models". Researchers should explicitly list CNNs, LSTMs, XGBoost, ablation models, and any other specific models they refer to.

3. Even though they provide a good context for wildfire impacts, machine learning approaches and the need for integrated fuel-climate modelling, they do not clearly articulate the research gap. They can add a paragraph to the introduction that explicitly states the limitations of current ML wildfire prediction, the lack of physics-informed deep learning models, and the absence of multi-scale temporal modelling.

4. It will be a good idea to include the datasets used, performance metrics and the limitations of the citations used under the literature review so that the section can have a bit more depth.

5. An additional transformer description will help the depth of the paper. Add attention equation, positional encoding and architecture details.

6. On the model architecture, some critical information appears missing, including the number of GRU layers, hidden units, transformer heads, embedding dimension, dropout rate, optimiser, learning rate and batch size. Without these, reproducibility will be impossible.

7. There is an inconsistent data time range "18 years of data (2005-2012)". This will affect the whole interpretation of data and should be rectified.

8. The datasize is also unclear. The table shows soil observations of 4458, climate 216, moisture 168,962 and then a final dataset of 33,560 samples. Either they are explained, or the differences are made clear.

9. The paper claims "21 features selected based on correlation" but they do not specify the threshold, method or dimensionality reduction. They should also apply KNN imputation only on the training data and not the entire set so that data leakage doesn't occur.

10. "with a time span of 30 days" makes it unclear whether the prediction horizon is 30 days or the training window is 30 days. Please clarify. Though the models used are listed, there is no information on hyperparameters, architecture and tuning procedure, and this may impact the fairness of the comparison.

11. Professional proofreading is recommended as some phrases give unintended meanings and make reading the paper a bit tedious.

**********

what does this mean?). If published, this will include your full peer review and any attached files.

If you choose “no”, your identity will remain anonymous but your review may still be made public.

**Do you want your identity to be public for this peer review?**  For information about this choice, including consent withdrawal, please see our Privacy Policy

Reviewer #1: No

Reviewer #2: No

**********

[NOTE: If reviewer comments were submitted as an attachment file, they will be attached to this email and accessible via the submission site. Please log into your account, locate the manuscript record, and check for the action link "View Attachments". If this link does not appear, there are no attachment files.]

To ensure your figures meet our technical requirements, please review our figure guidelines: https://journals.plos.org/plosone/s/figures

You may also use PLOS’s free figure tool, NAAS, to help you prepare publication quality figures: https://journals.plos.org/plosone/s/figures#loc-tools-for-figure-preparation.

NAAS will assess whether your figures meet our technical requirements by comparing each figure against our figure specifications.

Attachment Submitted filename: Reviewer Report.pdf

# Author comment: Author response to Decision Letter 1

**DOI:** 10.1371/journal.pone.0355829.r002

19 Apr 2026

Response to Reviewers

Dear Editor and Reviewers,

On behalf of all authors of this manuscript (Manuscript ID: PONE-D-26-00599), we sincerely thank the editorial team and the anonymous reviewers for their insightful comments and constructive suggestions, which have greatly improved the quality and clarity of the paper. We have carefully addressed each point in the revised manuscript. The comments fall into three categories: Key Revision Suggestions (10 questions), Reviewer #1 Report (6 questions), and Reviewer #2 Report (11 questions). Below is our point-by-point response. All revisions are marked in red. Should you have any further questions, please do not hesitate to contact us. We hope that the revised manuscript now meets the publication standards of your journal.

Key Revision Suggestions

1. The manuscript proposes a deep learning framework; however, the source code and access to this framework are not provided. Please provide the source code of the developed framework for journal readers. You may upload it to a repository or upload center and provide its link in the methodology section.

Author’s Reply:

Thank you for this important suggestion. We fully agree that sharing source code is essential for reproducibility. Accordingly, we have added the GitHub link (https://github.com/CBJYB/FWIMSNet-Forest-Fire-Risk-Prediction-Framework) to the framework source code in Section 2.5 (Methodology) on page 11. The repository includes a complete README and demo scripts to assist readers in using the framework.

2.Restructure the methodology based on the provided source code to present a step-by-step guideline for journal readers showing how to use this framework.

Author’s Reply:

Thank you for this constructive suggestion. In the revised manuscript, we have restructured the methodology section and added a step-by-step guideline in Section 2.5 (Methodology, starting on page 11), following the workflow of the source code. The overall execution flow includes data preprocessing (standardization and data augmentation), multi-scale input feature construction, model construction (multi-scale feature extraction, GRU-Transformer collaborative mechanism, and output layer), model training and evaluation, and finally prediction and evaluation. These steps are described in detail to help readers easily understand and use the proposed framework.

3.Sections 2 and 3 can be merged into a single section titled Methodology.

Author’s Reply:

Thank you for this helpful suggestion. In the revised manuscript, we have merged the original Sections 2 and 3 into a single Methodology section (now Section 2, beginning on page 6) and updated the section title accordingly. The subsections have also been renumbered for clarity.

4.The Introduction requires substantial revision in terms of both quality and quantity. Please include a more comprehensive literature review, clearly define the research gaps, and then highlight the novelty of the current study.

Author’s Reply:

Thank you for this constructive suggestion. We have substantially revised the Introduction (Section 1, starting from page 2) to address the concerns regarding both quality and quantity. Specifically:

More comprehensive literature review: We have systematically reviewed traditional sensor‑based methods, machine learning approaches, deep learning models, and physically‑enhanced methods. The revised Introduction now cites up‑to‑date works (references [5]–[16]) covering WSNs, random forest, gradient boosting, SVM, CNN‑BiLSTM, LSTNet, Transformer, enhanced FWI, and GIS‑based risk mapping.

Clearly defined research gaps: After the literature review, we explicitly point out three critical gaps: (1) most deep learning models treat fire prediction as a purely data‑driven task, overlooking the inherent multi‑scale temporal structure of fire physics; (2) the FWI system’s potential as a physically aligned organizational principle for feature engineering remains largely untapped; (3) existing architectures struggle to capture multi‑scale temporal patterns (daily to seasonal) and long‑term dependencies simultaneously.

Highlighted novelty: In response to the identified gaps, we list the three main contributions of our study: physically aligned feature organization, multi‑scale feature extraction, and temporal‑global fusion (GRU‑Transformer).

In addition, we have added a new Figure 1 with a data‑driven analysis of key fire indicators from the China Statistical Yearbook (2000–2023) to strengthen the motivation.

New references added in the revised Introduction (compared to the original version):

[5] (WSN real‑time monitoring), [6] (sensor types), [7] (early alarm), [8] (WSN challenges), [9] (Khurram et al., random forest & GBM), [10] (Chen et al., SVM), [11] (Škurić Kuraži et al., soil‑based index), [12] (Marjani et al., CNN‑BiLSTM), [13] (Lin et al., LSTNet), [14] (Miao et al., window‑enhanced Transformer), [15] (Santos et al., enhanced FWI), [16] (Kumari et al., GIS‑based risk).

We are grateful for the reviewer’s insightful suggestions, which have significantly improved the comprehensiveness and depth of our Introduction.

5. The methodology section (currently Sections 2 and 3) needs better organization. Please restructure it based on the newly added source code. These sections also require more supporting references. In addition, each subsection should specify which packages or libraries were used.

Author’s Reply:

Thank you for these valuable suggestions. In the revised manuscript, we have thoroughly restructured the methodology section based on the workflow of the newly added source code. First, we merged the original Sections 2 and 3 into a single Section 2 (Methodology) starting from page 6, following the logical flow: Forest Fire Weather Index → 1D-CNN → GRU → Transformer → Improved Model (FWI‑MSNet) → Data Preprocessing → Multi‑scale Input Construction → Multi‑scale Feature Extraction Module → GRU‑Transformer Collaborative Mechanism. Second, we added more supporting references throughout the section: for the FWI equations (Refs. [23–29] in Section 2.1), 1D-CNN (Refs. [30–35] in Section 2.2), GRU (Refs. [36–41] in Section 2.3), Transformer (Refs. [42–45] in Section 2.4), and the collaborative mechanism (Refs. [46–49] in Section 2.5.4). All equations now have appropriate citations.

Third, we have clearly specified the packages and libraries used in each subsection. Section 2.5 states Python 3.9, PyTorch 1.10, NumPy, Pandas, and scikit‑learn; Section 2.5.1 mentions sklearn.preprocessing.StandardScaler; Section 2.5.2 notes Pandas and scikit‑learn’s StandardScaler; Section 2.5.3 specifies torch.nn for Conv1d, BatchNorm1d, ReLU, and Dropout; and Section 2.5.4 provides detailed hyperparameter tables (e.g., Table 5) reflecting the PyTorch implementations of GRU and Transformer. In addition, all key model parameters and architectural configurations (e.g., kernel sizes, hidden dimensions, number of attention heads, etc.) have been supplemented in Tables 4 and 5 within the corresponding subsections, further enhancing reproducibility. All changes are marked in red.

We are grateful for the reviewer’s insightful suggestions, which have significantly improved the comprehensiveness and depth of our Introduction.

6. Ensure that each equation is supported with relevant references.

Author’s Reply:

Thank you for this important requirement. In the revised manuscript, we have added a reference at the beginning of each formula block. Specifically: reference [29] supports Equations (1)–(7) in Section 2.1; [35] supports Equation (8) in Section 2.2; [41] supports Equations (9)–(12) in Section 2.3; [45] supports Equations (13)–(18) in Section 2.4; and [49] supports Equation (19), [50] supports Equation (20) in Section 2.5.3.

For Equations (21)–(25), which include Z‑score normalization and the evaluation metrics RMSE, MAE, R², and MAPE, these are standard formulas widely available in the literature. To maintain conciseness, we have not cited specific references for them in the manuscript.

We are grateful for the reviewer’s insightful suggestions, which have significantly improved the comprehensiveness and depth of our Introduction.

7. For Table 6, please provide all the evaluation metrics separately for the training and testing datasets.

Author’s Reply:

Thank you for this valuable suggestion. In the revised manuscript, we have provided the evaluation metrics separately for the training and testing datasets. Specifically, Table 8 (originally Table 6, but renumbered due to newly added tables earlier in the manuscript) presents all metrics for the training set, and Table 9 presents all metrics for the test set. Both tables are located in Section 4.3 (pages 31–32).

We are grateful for the reviewer’s insightful suggestions, which have significantly improved the comprehensiveness and depth of our Introduction.

8. Add a Discussion section and mention the limitations of the current study.

Author’s Reply:

Thank you for this important suggestion. In the revised manuscript, we have added a new Discussion section (now Section 5, starting on page 36), which explicitly discusses the limitations of the current study and outlines future research directions. Specifically, we point out three main limitations: (1) the model is trained only on data from a single ecological station in China, lacking spatial information such as vegetation indices and topographic features; (2) the current architecture does not model spatial fire propagation and has high computational complexity; (3) the Transformer’s self‑attention mechanism is not fully interpretable, and the model does not output intermediate physical states. Corresponding future work directions are also provided.

9. The Conclusion needs improvement. Please clearly list the key findings of the study in the conclusion.

Author’s Reply:

Thank you for this constructive suggestion. In the revised manuscript, we have thoroughly rewritten the Conclusion section (now Section 6, starting on page 37) to clearly list the key findings of the study. Specifically, we summarize five main findings: (1) superior prediction performance with detailed quantitative metrics (RMSE, MAE, R², MAPE); (2) effectiveness of physical alignment via FWI-based feature grouping; (3) multi-scale feature extraction using parallel 1D‑CNN with kernel sizes 7, 30, and 90; (4) temporal‑global fusion achieved by the GRU‑Transformer collaborative mechanism; and (5) case study validation on the 2020 Australian wildfires demonstrating generalizability.

We are grateful for the reviewer’s insightful suggestions, which have significantly improved the comprehensiveness and depth of our Introduction.

10. Each table and figure in the Results section requires more complete explanation and discussion in the text.

Author’s Reply:

Thank you for this important suggestion. In the revised manuscript, we have carefully reviewed every table and figure and added more complete explanations and discussions in the corresponding text. Specifically, we have expanded the description and analysis for Table 1 (page 6, Section 1), Table 2 (page 12, Section 2.5.2), Table 3 (page 13, Section 2.5.2), Table 4 (page 14, Section 2.5.3), Table 5 (page 19, Section 2.5.4), Table 6 (page 20, Section 3.1), Table 7 (page 24, Section 3.3), Table 8 (page 31, Section 4.3), Table 9 (page 32, Section 4.3), Table 10 (page 35, Section 4.4), as well as for Figure 1 (page 2, Section 1), Figure 2 (page 10, Section 2.5), Figure 3 (page 16, Section 2.5.3), Figure 4 (page 17, Section 2.5.4), Figure 5 (page 22, Section 3.3), Figure 6 (page 27, Section 4.2), Figure 7 (page 29, Section 4.3), Figure 8 (page 30, Section 4.3), Figure 9 (page 33, Section 4.3), and Figure 10 (page 34, Section 4.4). Each table and figure is now accompanied by a thorough discussion that highlights key observations, implications, and connections to the research objectives.

We are grateful for the reviewer’s insightful suggestions, which have significantly improved the comprehensiveness and depth of our Introduction.

Reviewer #1:Reviewer Report

Recommendation

The topic is important and the pipeline is reasonable, but several key claims and evaluation details limit confidence. I recommend major revision to strengthen (a) the definition of the prediction task and inputs construction, (b) training leakage control, and (c) “physics-informed” claims in the title.

Author’s Reply:

We thank the reviewer for summarizing the key concerns. We have carefully addressed all three points—(a) prediction task definition and input construction, (b) training leakage control, and (c) the “physics‑informed” claim—in the revised manuscript. Detailed point‑by‑point responses are provided below.

1. “Physics-informed” is currently not demonstrated in the standard sense: The manuscript positions FWI as “the physical core” and describes the framework “physics-informed”. But the model appears to be a data-driven architecture guided by feature grouping rather than physics-informed optimization. The model does not explicitly incorporate physical constraints or differentiable governing equations. Please either rename the approach or add a true physics-informed component.

Author’s Reply:

We sincerely thank the reviewer for this critical and insightful comment. We fully agree that the original use of the term “physics-informed” was inappropriate, as our model does not embed differentiable physical constraints or governing equations in the sense of Physics-Informed Neural Networks (PINNs). To avoid any misunderstanding, we have thoroughly revised the manuscript as follows:

First, we have renamed the approach throughout the paper. The new title is:

“Physically aligned Forest Fire Risk Prediction: A Deep Learning Framework Coupling Fuel and Climate Multivariate Factors”

All instances of “physics-informed” have been replaced with more accurate terms such as “physically aligned”, “feature‑guided”, or “data‑driven with physical feature grouping”. The term “physics‑informed” no longer appears in the manuscript.

Second, we have clarified that the model is aligned with physical principles through its architecture and feature organization, rather than through explicit differentiable constraints. Specifically:

The input features are grouped according to the multi‑scale temporal structure of the FWI system (short‑term climate‑driven, medium‑term eco‑hydrological, long‑term soil‑driven).

The kernel sizes of the parallel 1D‑CNN channels (7, 30, 90 days) are explicitly mapped to the characteristic timescales of FFMC, DMC, and DC dynamics, respectively.

The GRU‑Transformer collaborative mechanism captures both sequential dependencies and global contextual correlations, which are physically interpretable as fuel‑moisture evolution and climate‑anomaly modulation.

We believe that “physically aligned” accurately reflects the design philosophy of the proposed framework without overclaiming a true PINN implementation. All changes have been marked in red in the revised manuscript (see Title, Abstract, Introduction, and Section 2). We are grateful to the reviewer for helping us improve the terminological precision of our paper.

We are grateful for the reviewer’s insightful suggestions, which have significantly improved the comprehensiveness and depth of our Introduction.

2. High risk of leakage in evaluation: The paper reports a random stratified split into train/val/test (70/15/15). For time series data this can introduce temporal leakage. Also, the model uses multi-scale convolutions with long-term path (up to a quarter), which could cause leakage. Please ensure that no overlapping windows cross split boundaries.

Author’s Reply:

We thank the reviewer for raising this critical issue. We apologize for the confusion caused by the earlier description of the data split (70/15/15 random stratified split), which was a writing error and did not reflect the actual implementation. In fact, we used a chronological split of 80% training / 20% testing (no validation set was used separately). This has been corrected in the revised manuscript. Specifically, we have added a detailed description in Section 3.2 (page 21) as follows:

(1) Chronological split (no leakage): The first 80% of time‑ordered samples are used for training and the last 20% for testing. No future information

Attachment Submitted filename: Response to Reviewers.docx

# Peer review: Decision Letter 1

**DOI:** 10.1371/journal.pone.0355829.r003

**Authors:** Julia A. Jones

18 Jun 2026

Dear Dr. Zeng,

Thank you for submitting your manuscript to PLOS ONE. After careful consideration, we feel that it has merit but does not fully meet PLOS ONE’s publication criteria as it currently stands. Therefore, we invite you to submit a revised version of the manuscript that addresses the points raised during the review process.

**See my comments below.**

Please submit your revised manuscript by Aug 02 2026 11:59PM. If you will need more time than this to complete your revisions, please reply to this message or contact the journal office at plosone@plos.org. When you're ready to submit your revision, log on to https://www.editorialmanager.com/pone/ and select the 'Submissions Needing Revision' folder to locate your manuscript file.

- A letter that responds to each point raised by the academic editor and reviewer(s). You should upload this letter as a separate file labeled 'Response to Reviewers'.
- A marked-up copy of your manuscript that highlights changes made to the original version. You should upload this as a separate file labeled 'Revised Manuscript with Track Changes'.
- An unmarked version of your revised paper without tracked changes. You should upload this as a separate file labeled 'Manuscript'.

If you would like to make changes to your financial disclosure, please include your updated statement in your cover letter. Guidelines for resubmitting your figure files are available below the reviewer comments at the end of this letter.

If applicable, we recommend that you deposit your laboratory protocols in protocols.io to enhance the reproducibility of your results. Protocols.io assigns your protocol its own identifier (DOI) so that it can be cited independently in the future. For instructions see: https://journals.plos.org/plosone/s/submission-guidelines#loc-laboratory-protocols. Additionally, PLOS ONE offers an option for publishing peer-reviewed Lab Protocol articles, which describe protocols hosted on protocols.io. Read more information on sharing protocols at https://plos.org/protocols?utm_medium=editorial-email&utm_source=authorletters&utm_campaign=protocols.

As the corresponding author, your ORCID iD is verified in the submission system and will appear in the published article. PLOS supports the use of ORCID, and we encourage all coauthors to register for an ORCID iD and use it as well. Please encourage your coauthors to verify their ORCID iD within the submission system before final acceptance, as unverified ORCID iDs will not appear in the published article. *Only*  the individual author can complete the verification step; PLOS staff *cannot*  verify ORCID iDs on behalf of authors.

We look forward to receiving your revised manuscript.

Kind regards,

Julia A. Jones

Academic Editor

PLOS One

**Journal Requirements:**

If the reviewer comments include a recommendation to cite specific previously published works, please review and evaluate these publications to determine whether they are relevant and should be cited. There is no requirement to cite these works unless the editor has indicated otherwise.

**Additional Editor Comments:**

The paper does not meet the PLOS One criteria for papers that describe new methods or software for applications.  There are also some concerning omissions in the paper. Overall, it is unclear how, or why, the model is useful for fire risk prediction.

Omissions in the paper:

(1) key datasets are not provided

(2) important variables used in the model are not defined or explained

(3) the insights provided by deep learning are not compared to other model approaches

(4) key literature citations are unpublished reports

(5) The third reviewer raises issues, which were also raised by the first two reviewers.  In their revision, the authors did not address the key questions of all reviewers, which are, what does this model (deep learning) with a fire weather index contribute, and why does it improve fire risk predictions?

(6) The figures are of poor quality.

Failure to meet PLOS One criteria for papers that describe new methods or software for applications:

(1) utility: PLOS One requirement: “The tool must be of use to the community and must present a proven advantage over existing alternatives, where applicable.“

The model presented in the paper performs well because of the incorporation of a fire weather index. However, the manuscript does not explain how the fire weather index is calculated, why deep learning using the fire weather index improves model performance, or how their use of the fire weather index in deep learning is different from other models. The fire weather index citations are two unpublished reports from Canada. It is not explained how field data were used to create a fire weather index.

(2) validation: PLOSone requires that ““Submissions presenting methods, software, databases, or tools must demonstrate that the new tool achieves its intended purpose. If similar options already exist, the submitted manuscript must demonstrate that the new tool is an improvement over existing options in some way. This requirement may be met by including a proof-of-principle experiment or analysis;”

A validation is performed for a case study of fire in Australia in 2020, but it is not explained. No citation is provided for the data used for the case study in Australia, either in the literature cited or the data availability statement. The validation results are shown in Figure 10 but only as a fire weather index, and the calculation of the fire weather index is not defined or explained in the paper.

(3) availability: PLOS One requirement: “If the manuscript’s primary purpose is the description of new software or a new software package, this software must be open source, deposited in an appropriate archive, and conform to the Open Source Definition. “

The data availability statement does not provide any links to data. The model software is not available. There are no links provided to key datasets, such as the fire weather index, which was central to their model. The only datasets provided are for meteorological, water, and soil data from the National Research Station of Huitong Forest Ecosystems and some ERA5 data.

[Note: HTML markup is below. Please do not edit.]

Reviewers' comments:

Reviewer's Responses to Questions

**Comments to the Author**

Reviewer #2: All comments have been addressed

Reviewer #3: All comments have been addressed

Reviewer #4: All comments have been addressed

**********

2. Is the manuscript technically sound, and do the data support the conclusions?

Reviewer #2: Yes

Reviewer #3: No

Reviewer #4: Yes

**********

3. Has the statistical analysis been performed appropriately and rigorously?

Reviewer #2: Yes

Reviewer #3: No

Reviewer #4: Yes

**********

4. Have the authors made all data underlying the findings in their manuscript fully available?

The PLOS Data policy

Reviewer #2: Yes

Reviewer #3: (No Response)

Reviewer #4: Yes

**********

5. Is the manuscript presented in an intelligible fashion and written in standard English?

Reviewer #2: Yes

Reviewer #3: No

Reviewer #4: Yes

**********

**Reviewer #2:**  I can see extensive work has been done, and the document has been considerably improved. The Dataset has now been made available, and the abstract and methodology sections have been revised as advised. References have also been updated with more current literature.

**Reviewer #3:**  1. The writing of the paper needs a lot of improvement in terms of grammar, spelling, and presentation.

2. What is the novelty of this paper?

3. Add a flowchart to show what is done in this study.

4. Most Figures are blurred.

5. The mathematics used throughout the article is still not very strict. Please try to update and illustrate some elements in the mathematical model that are not defined very strictly.

6. The result part is week, results and discussion should be better explained.

7. Revise the conclusions and make it concise, (Better to write the main outcomes)

8. Include these Key Suggested References:

https://doi.org/10.1038/s41598-025-14908-x

DOI 10.21608/SVUSRC.2024.308832.1232

https://doi.org/10.58491/2735-4202.3263

https://doi.org/10.1038/s41598-025-16828-2

https://doi.org/10.1038/s41598-025-87208-z

**Reviewer #4:**  (No Response)

**********

what does this mean?). If published, this will include your full peer review and any attached files.

If you choose “no”, your identity will remain anonymous but your review may still be made public.

**Do you want your identity to be public for this peer review?**  For information about this choice, including consent withdrawal, please see our Privacy Policy

Reviewer #2: No

Reviewer #3: No

Reviewer #4: **Yes:** Pengjiao Jia

**********

[NOTE: If reviewer comments were submitted as an attachment file, they will be attached to this email and accessible via the submission site. Please log into your account, locate the manuscript record, and check for the action link "View Attachments". If this link does not appear, there are no attachment files.]

To ensure your figures meet our technical requirements, please review our figure guidelines: https://journals.plos.org/plosone/s/figures

You may also use PLOS’s free figure tool, NAAS, to help you prepare publication quality figures: https://journals.plos.org/plosone/s/figures#loc-tools-for-figure-preparation.

NAAS will assess whether your figures meet our technical requirements by comparing each figure against our figure specifications.

# Author comment: Author response to Decision Letter 2

**DOI:** 10.1371/journal.pone.0355829.r004

19 Jul 2026

Response to Reviewers

Dear Editor and Reviewers,

We sincerely thank the Academic Editor and all reviewers for their thoughtful and constructive comments on our manuscript. We have carefully addressed each concern raised and provide a point‑by‑point response below. All changes in the revised manuscript have been highlighted for easy identification.

In response to the Additional Editor Comments, we have made the following key improvements: (1) added a Data Availability Statement with DOIs for the three source datasets, the GitHub repository link, and the ERA5 data source; (2) supplemented definitions for all key model variables and hyperparameters; (3) added a new subsection (Section 4.5) to explicitly compare the scientific insights of our deep learning framework with alternative models; (4) added two peer‑reviewed references (Wotton, 2009; Kudláčková et al., 2024) to supplement the original technical report; (5) clarified the model‘s contribution and why it improves predictions in Section 4.5; and (6) improved all figures using the NAAS tool to meet PLOS ONE’s technical requirements.

We are grateful to the editor for pointing out the figure numbering issue in the case study figure caption of Section 4.4. In response, we have corrected the figure number in the caption from “Fig 1” to “Fig 11” to match the in‑text citation.

We have also responded to all reviewer comments, including those from Reviewer #2 (positive feedback), Reviewer #3 (eight specific points), and Reviewer #4 (no specific comments). All revisions have been made to the manuscript accordingly. We believe the revised version now meets the publication standards of PLOS ONE.

Additional Editor Comments:

Omissions in the paper:

1. key datasets are not provided.

Author’s Reply:

We thank the editor for this valuable suggestion. In the revised manuscript, we have added a dedicated Data Availability Statement (placed before the References) that clearly:

(1) lists the three source datasets with their DOI links from the China Ecological Science Data Center;

(2) explains that the full datasets are subject to third-party data usage agreements and are accessible through the data center;

(3) provides the link to our GitHub repository, which contains the complete source code and a demo dataset for readers to test the model;

(4) cites the ERA5 data source for the case study.

We believe these additions fully address the data availability concern. We sincerely appreciate the editor’s time and thoughtful guidance in helping us improve the transparency and reproducibility of our work.

2. important variables used in the model are not defined or explained.

Author’s Reply:

We thank the reviewer for this valuable comment. Upon careful review, we found that most of the key variables and hyperparameters used in our model were already defined and explained in the original manuscript. However, we also identified that the data preprocessing parameters in Table 2 lacked explicit justification. To address this, we have added a dedicated paragraph after Table 2 to explain these parameters, including the choice of sequence length (6 months), stride (1), noise levels (0.01 and 0.02), and the 80/20 chronological split.

Specifically:

FWI system variables (FFMC, DMC, DC, ISI, BUI, FWI) and their physical meanings were already provided in Section 2.1.

Multi-scale CNN parameters (kernel sizes 7, 30, 90 and out_channels 32) were already explained in Section 2.5.3.

GRU-Transformer hyperparameters (hidden_dim, nhead, num_layers, etc.) were already explained in Section 2.5.4.

Data preprocessing parameters (base_seq_length, stride, noise_levels, train_ratio, test_ratio) — we have now added explicit explanations for these in a new paragraph after Table 2.

We believe these additions and clarifications fully address the reviewer's concern. We sincerely appreciate the reviewer’s valuable suggestion, which has helped us improve the clarity of our manuscript.

3. the insights provided by deep learning are not compared to other model approaches.

Author’s Reply:

We thank the reviewer for this constructive suggestion. In the revised manuscript, we have added a new subsection in the Discussion (Section 4.5) that explicitly compares the scientific insights derived from our deep learning framework with those from alternative model approaches.

Specifically, based on the experimental results reported in Table 9, we highlight three key insights revealed by FWI-MSNet that traditional models (e.g., XGBoost, LSTM, CNN) cannot provide:

(1) The FWI system serves as a critical physical anchor for fire risk prediction. Removing FWI features degrades R² from 0.9251 to 0.5115 (a 44.7% decrease relative to the full model), demonstrating that the FWI system is not merely an additional input variable but a foundational physical prior. Traditional models that treat all inputs uniformly cannot reveal this distinction.

(2) The simultaneous modeling of daily, monthly, and seasonal scales is essential. Removing multi-scale convolution increases RMSE from 1.6892 to 2.4227 (a 43.4% increase, based on Table 9). This indicates that the fuel-climate coupling mechanism operates across multiple temporal scales simultaneously, and a single-scale model inevitably misses critical information at other scales.

(3) GRU and Transformer play distinct but complementary roles. Removing GRU increases RMSE to 2.4466, while removing Transformer increases it to 3.0047 (Table 9). This decomposition reveals that GRU primarily captures local sequential evolution of fuel moisture, whereas Transformer captures global cross-period correlations (e.g., remote modulation by climate anomalies).

We believe these additions, derived directly from the ablation experiments in Table 9, demonstrate that our physically aligned deep learning framework offers unique interpretable insights into the multi-scale fuel-climate coupling mechanism—a capability that conventional black-box models lack. We sincerely appreciate the reviewer's insightful suggestion, which has helped us better articulate the unique value of our deep learning framework and strengthened the overall discussion of our findings. We believe this addition fully addresses the reviewer's concern.

4. key literature citations are unpublished reports.

Author’s Reply:

We thank the reviewer for this careful observation. The reviewer is referring to Ref. [29] (Van Wagner & Pickett, 1985), which is a Technical Report from the Canadian Forestry Service. We cited this report because it is the original and definitive source that documents the complete mathematical formulation of the FWI system—the core physical foundation of our model. This report remains the standard reference for FWI equations in the fire research community.

However, we agree with the reviewer that supplementing this technical report with peer-reviewed literature strengthens the paper. In the revised manuscript, we have added two peer-reviewed references on the FWI system:

Wotton, B.M. (2009) — Interpreting and using outputs from the Canadian Forest Fire Danger Rating System in research applications. Environmental and Ecological Statistics, 16(2), 107–131. This reference provides a comprehensive, peer-reviewed overview of the FWI system and its outputs, serving as an authoritative supplement to the original technical report.

Kudláčková, L., et al. (2024) — Assessing fire danger classes and extreme thresholds of the Canadian Fire Weather Index across global environmental zones: a review. Environmental Research Letters, 20(1), 013001. This recent review synthesizes findings from 750 studies across 18 global environmental zones, demonstrating the widespread applicability and effectiveness of the FWI system.

Both references have been cited in Section 2.1 alongside the original Technical Report [29], ensuring that readers have access to both the foundational mathematical formulation and supporting peer-reviewed evidence. We sincerely appreciate the reviewer's valuable suggestion, which has helped us strengthen the literature foundation of our manuscript.

5. The third reviewer raises issues, which were also raised by the first two reviewers. In their revision, the authors did not address the key questions of all reviewers, which are, what does this model (deep learning) with a fire weather index contribute, and why does it improve fire risk predictions?

Author’s Reply:

We sincerely thank the editor for this critical comment. To directly address the key questions raised by all reviewers—what does this model contribute, and why does it improve fire risk predictions?—we have added a new subsection in the Discussion (Section 4.5) titled “Insights from Deep Learning Compared to Alternative Models.”

In this subsection, we explicitly articulate that our contribution lies not in proposing a new deep learning architecture, but in establishing a physically aligned framework that uses the FWI system as an organizational principle for feature engineering. Specifically, we demonstrate three key insights based on the quantitative results reported in Table 9:

The FWI system provides a critical physical anchor. Removing FWI features degrades R² from 0.9251 to 0.5115, a 44.7% decrease.

Multi-scale modeling is essential. Removing multi-scale convolution increases RMSE from 1.6892 to 2.4227, a 43.4% increase.

GRU and Transformer play distinct but complementary roles. Removing GRU increases RMSE to 2.4466, while removing Transformer increases it to 3.0047.

These findings demonstrate that FWI-MSNet improves predictions by aligning the model architecture with the multi-scale physical structure of the fuel-climate coupling mechanism—an approach that conventional black-box models (XGBoost, LSTM, CNN) cannot provide. We have added this new subsection to the revised manuscript (Section 4.5). We believe this addition fully addresses the reviewers' concern. We sincerely appreciate the editor's and reviewers' valuable guidance, which has helped us significantly improve the clarity of our contribution.

6. The figures are of poor quality.

Author’s Reply:

We thank the editor for raising this concern. In the revised manuscript, we have thoroughly improved all figures to meet PLOS ONE's technical requirements.

Specifically, all eleven figures were uploaded to and checked using the NAAS digital diagnostic tool (https://ngplosjournals.pagemajik.ai/artanalysis), the official pre-submission image quality checker recommended by PLOS ONE. The tool confirmed that ten of the eleven figures passed the inspection and were adjusted to meet the specifications, including resolution adjustment to 300 DPI where needed, dimensional adjustment to within the required width limit (≤ 7.5 inches), and conversion to valid TIFF format. For Figure 7, the tool indicated that it could not automatically adapt the image; we manually adjusted its width to 7.5 inches with 300 DPI resolution and re-exported it as a TIFF file. The corrected version now fully complies with PLOS ONE's specifications.

We believe all figures now meet the publication standards of PLOS ONE. We sincerely appreciate the editor's attention to this important presentation detail.

Failure to meet PLOS One criteria for papers that describe new methods or software for applications:

1. utility: PLOS One requirement: “The tool must be of use to the community and must present a proven advantage over existing alternatives, where applicable.“

The model presented in the paper performs well because of the incorporation of a fire weather index. However, the manuscript does not explain how the fire weather index is calculated, why deep learning using the fire weather index improves model performance, or how their use of the fire weather index in deep learning is different from other models. The fire weather index citations are two unpublished reports from Canada. It is not explained how field data were used to create a fire weather index.

Author’s Reply:

We thank the editor for this comment. We have carefully addressed each of the three concerns raised.

First, regarding the calculation of the FWI: The FWI system and its complete mathematical formulations are already provided in Section 2.1 (Equations 1–7), where all six components (FFMC, DMC, DC, ISI, BUI, and FWI) are defined with their governing equations. Additionally, Section 3.2 describes how the FWI components were calculated from the field observations (temperature, relative humidity, wind speed, and precipitation) on a daily basis. Regarding the citation: we have retained Ref. [29] (Van Wagner & Pickett, 1985) as the original source of the FWI equations, and have supplemented it with two peer-reviewed references [56, 57] in the revised manuscript.

Second, regarding why deep learning with FWI improves performance: We have added a new subsection in the Discussion (Section 4.5) that explicitly explains the mechanisms. Based on the ablation results in Table 9, we demonstrate that the FWI system serves as a physical anchor—removing FWI features degrades R² from 0.9251 to 0.5115 (a 44.7% decrease). This confirms that the FWI system is not merely an additional input but a foundational physical prior that constrains and guides the learning process.

Third, regarding how our use of FWI differs from other models: We have clarified this in both the Introduction and Section 4.5. Unlike conventional approaches that treat FWI components merely as additional input variables, our framework uses the FWI system as an organizational principle for feature engineering. Specifically, we explicitly map the FWI‘s inherent multi‑scale temporal structure (FFMC—daily, DMC—monthly, DC—seasonal) to the parallel 1D‑CNN kernel sizes (7, 30, and 90 days). This ensures that the learning process operates at the same temporal scales as the physical processes governing fire risk formation—a design that conventional black‑box models lack.

We believe these clarifications fully address the editor’s concerns. We sincerely appreciate the editor’s valuable guidance, which has helped us better articulate the utility of our framework.

2. validation: PLOSone requires that ““Submissions presenting methods, software, databases, or tools must demonstrate that the new tool achieves its intended purpose. If similar options already exist, the submitted manuscript must demonstrate that the new tool is an improvement over existing options in some way. This requirement may be met by including a proof-of-principle experiment or analysis;”

A validation is performed for a case study of fire in Australia in 2020, but it is not explained. No citation is provided for the data used for the case study in Australia, either in the literature cited or the data availability statement. The validation results are shown in Figure 10 but only as a fire weather index, and the calculation of the fire weather index is not defined or explained in the paper.

Author’s Reply:

We thank the editor for this comment. In the revised manuscript, we have made the following clarifications to address each of the concerns raised.

First, regarding the citation for the Australian case study data: The ERA5 reanalysis data used for the case study was already cited as Ref. [55] in the original manuscript. In the revised version, we have also explicitly listed this dataset in the Data Availability Statement to ensure readers can clearly identify the data source.

Second, regarding the calculation of the FWI for the case study: The FWI calculation follows the same mathematical formulations already defined in Section 2.1 (Equations 1–7). To make this explicit, we have added the following sentence at the beginning of Section 4.4:

“The FWI values for the case study are calculated using the same mathematical formulations described in Section 2.1, driven by ERA5 meteorological inputs (temperature, relative humidity, wind speed, and precipitation).”

Third, regarding the case study description: The case study setup, including the six representative regions across Australia (East Gippsland, Canberra, Blue Mountains, Melbourne, South Coast, and Sydney Area), is already described in Section 4.4. The validation results are quantitatively reported in Table 10, with R² values ranging from 0.57 to 0.77 and MAPE approximately 25% across all regions. This demonstrates cross-regional generali

Attachment Submitted filename: Response_to_Reviewers_auresp_2.docx

# Editor report: Decision Letter 2

**DOI:** 10.1371/journal.pone.0355829.r005

**Authors:** Julia A. Jones

27 Jul 2026

Physically aligned Forest Fire Risk Prediction: A Deep Learning Framework Coupling Fuel and Climate Multivariate Factors

PONE-D-26-00599R2

Dear Dr. Zeng,

We’re pleased to inform you that your manuscript has been judged scientifically suitable for publication and will be formally accepted for publication once it meets all outstanding technical requirements.

Within one week, you’ll receive an e-mail detailing the required amendments. When these have been addressed, you’ll receive a formal acceptance letter and your manuscript will be scheduled for publication.

An invoice will be generated when your article is formally accepted. Please note, if your institution has a publishing partnership with PLOS and your article meets the relevant criteria, all or part of your publication costs will be covered. Please make sure your user information is up-to-date by logging into Editorial Manager at Editorial Manager® and clicking the ‘Update My Information' link at the top of the page. For questions related to billing, please contact billing support.

If your institution or institutions have a press office, please notify them about your upcoming paper to help maximize its impact. If they’ll be preparing press materials, please inform our press team as soon as possible -- no later than 48 hours after receiving the formal acceptance. Your manuscript will remain under strict press embargo until 2 pm Eastern Time on the date of publication. For more information, please contact onepress@plos.org.

Kind regards,

Julia A. Jones

Academic Editor

PLOS One

Additional Editor Comments (optional):

Thank you for your response to my questions regarding PLOS One requirements for this type of paper, and to the review comments from the reviewer of the R1 version.

Reviewers' comments:

# Editor report: Acceptance letter

**DOI:** 10.1371/journal.pone.0355829.r006

**Authors:** Julia A. Jones

PONE-D-26-00599R2

PLOS One

Dear Dr. Zeng,

I'm pleased to inform you that your manuscript has been deemed suitable for publication in PLOS One. Congratulations! Your manuscript is now being handed over to our production team.

At this stage, our production department will prepare your paper for publication. This includes ensuring the following:

* All references, tables, and figures are properly cited

* All relevant supporting information is included in the manuscript submission,

* There are no issues that prevent the paper from being properly typeset

You will receive further instructions from the production team, including instructions on how to review your proof when it is ready. Please keep in mind that we are working through a large volume of accepted articles, so please give us a few days to review your paper and let you know the next and final steps.

Lastly, if your institution or institutions have a press office, please let them know about your upcoming paper now to help maximize its impact. If they'll be preparing press materials, please inform our press team within the next 48 hours. Your manuscript will remain under strict press embargo until 2 pm Eastern Time on the date of publication. For more information, please contact onepress@plos.org.

You will receive an invoice from PLOS for your publication fee after your manuscript has reached the completed accept phase. If you receive an email requesting payment before acceptance or for any other service, this may be a phishing scheme. Learn how to identify phishing emails and protect your accounts at https://explore.plos.org/phishing.

If we can help with anything else, please email us at customercare@plos.org.

Thank you for submitting your work to PLOS One and supporting open access.

Kind regards,

PLOS ONE Editorial Office Staff

on behalf of

Dr. Julia A. Jones

Academic Editor

PLOS One
